# Variable Dendritic Integration in Hippocampal CA3 Pyramidal Neurons

**DOI:** 10.1016/j.neuron.2013.10.033

**Published:** 2013-12-18

**Authors:** Judit K. Makara, Jeffrey C. Magee

**Affiliations:** 1 Institute of Experimental Medicine, Hungarian Academy of Sciences, 1083 Budapest, Hungary; 2 Howard Hughes Medical Institute, Janelia Farm Research Campus, Ashburn, VA 20147, USA

## Abstract

The hippocampal CA3 region is essential for pattern completion and generation of sharp-wave ripples. During these operations, coordinated activation of ensembles of CA3 pyramidal neurons produces spatiotemporally structured input patterns arriving onto dendrites of recurrently connected CA3 neurons. To understand how such input patterns are translated into specific output patterns, we characterized dendritic integration in CA3 pyramidal cells using two-photon imaging and glutamate uncaging. We found that thin dendrites of CA3 pyramidal neurons integrate synchronous synaptic input in a highly supralinear fashion. The amplification was primarily mediated by NMDA receptor activation and was present over a relatively broad range of spatiotemporal input patterns. The decay of voltage responses, temporal summation, and action potential output was regulated in a compartmentalized fashion mainly by a G-protein-activated inwardly rectifying K^+^ current. Our results suggest that plastic dendritic integrative mechanisms may support ensemble behavior in pyramidal neurons of the hippocampal circuitry.

## Highlights

• Active nonlinear dendritic integration in CA3 pyramidal neurons
• NMDARs mediate amplification of synchronous synaptic inputs
• Compartmentalized control of dendritic integration and somatic output by K+ channels

## Teaser

Makara and Magee demonstrate NMDA receptor-mediated supralinear dendritic integration of multiple coactive synaptic inputs in thin dendrites of hippocampal CA3 pyramidal neurons and reveal a powerful regulation of these NMDA spikes by G-protein-activated inwardly rectifying K+ channels.

## Introduction

The dynamic formation of neuronal ensembles is thought to be fundamental for information encoding and storage in nervous systems. Although the cellular and network mechanisms leading to the formation of such neuronal population activity are poorly understood, it is generally assumed that synaptic plasticity among coactive neurons is primarily involved in the process. Recent studies shed light on another powerful neuronal mechanism that could play a role in enhancing coactivation of connected neurons. Active forms of dendritic integration, produced through dendritic voltage-dependent conductances (Magee and Johnston, 2005; Gulledge et al., 2005; Sjöström et al., 2008) may enable neurons to preferentially respond to the correlated firing of a neuronal ensemble (Losonczy and Magee, 2006; Remy et al., 2009; Branco et al., 2010) and the long-term modulation of active integration provides an additional mechanism to facilitate the generation and maintenance of ensemble activity (Magee and Johnston, 2005; Losonczy et al., 2008; Makara et al., 2009, Legenstein and Maass, 2011).

Spatiotemporally clustered input patterns may generate distinct types of dendritic nonlinearities in pyramidal neurons (Magee and Johnston, 2005; Gulledge et al., 2005; Sjöström et al., 2008; Larkum et al., 2009). Characteristic dendritic spike mechanisms include fast Na^+^ spikes and slow spikes mediated by NMDA receptors (NMDARs) and/or voltage-gated Ca^2+^ channels. Fast dendritic Na^+^ spikes are modulated by short-term as well as long-term plasticity in CA1PCs (Losonczy et al., 2008; Makara et al., 2009; Remy et al., 2009; Müller et al., 2012). Specifically, an NMDAR-dependent long-term potentiation of the propagation of Na^+^ spikes is expressed by the downregulation of Kv4.2 subunit containing K^+^ channel function (branch strength plasticity [BSP]; Losonczy et al., 2008; Makara et al., 2009). These studies open the door for exploring a new level of regulation of dendritic computation that concerns specifically the processing of information carried by activity of correlated cell groups.

The extensive recurrent collateral system (commissural/associational axons) connecting pyramidal cells in the hippocampal CA3 region (CA3PCs) is thought to promote the flexible formation and reorganization of information-coding ensembles. In fact, this property of CA3 is considered to be essential for autoassociative storage and recall of memory-related patterns (Marr, 1971; McNaughton and Morris, 1987; Rolls and Kesner, 2006) and for replaying sequences of previous activity patterns during sharp-wave ripples (SWRs) that promote memory consolidation (O’Neill et al., 2010). The recent evidence for pronounced spatiotemporal clustering of functionally related synapses in dendritic segments of CA3 pyramidal neurons (Kleindienst et al., 2011; Takahashi et al., 2012) implicates active dendritic integration as a potential component of ensemble formation in this region. However, although the basic anatomical, electrophysiological, and synaptic properties of rodent CA3 pyramidal cells have been characterized (e.g., Amaral and Witter, 1989; Li et al., 1994; Miles and Wong, 1986; Spruston et al., 1995; Urban and Barrionuevo, 1998), exploration of dendritic integration and plasticity has only recently begun (Kim et al., 2012). Here we set out to study the impact of active dendritic integration on the processing of correlated synaptic input such as that generated by ensemble activity in the CA3 circuitry.

## Results

### Supralinear Integration in Thin Dendrites of CA3 Pyramidal Neurons

**Figure 1:**
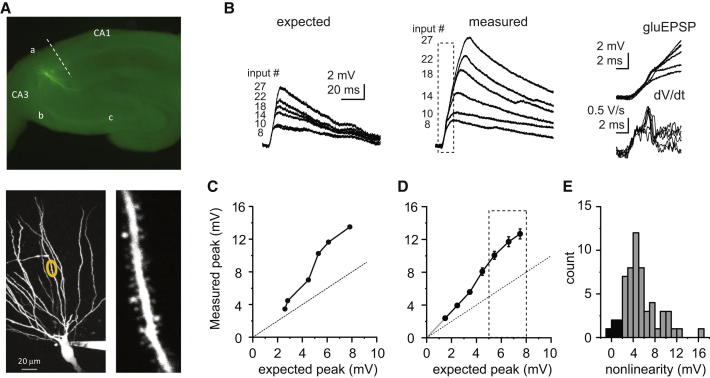
Nonlinear Dendritic Integration of Multiple Synchronous Synaptic Inputs in Basal Dendrites of CA3 Pyramidal Neurons (A) Top: low-magnification fluorescent image of a hippocampal slice with a CA3 pyramidal neuron loaded with Alexa Fluor 488. Bottom left: two-photon stack of the basal arborization of the same neuron. Dendritic segment of interest is highlighted by yellow circle. Bottom right: high-magnification stack of the dendritic segment. (B) Arithmetic sum (left) and measured responses (middle) to a varying number of synaptic inputs evoked by two-photon glutamate uncaging. Right: dotted box in middle panel expanded (top) with corresponding first temporal derivatives of the traces (bottom). (C) Input-output function of the dendrite in (B). Dotted line represents linear function. (D) Summary (mean ± SEM) of the input-output function of 57 branches from 42 cells. Dotted line represents linear function. Gray line represents linear projection of the slope of the measured points under 4 mV expected peak. Error bars for the x axis are within the symbols. (E) Histogram of the nonlinearity, measured as the average peak_gluEPSP_–peak_expected_ in the 5–8 mV expected amplitude range (dotted box in D). Dendrites with nonlinearity >2 mV (gray) were considered as supralinear integrators.

To investigate dendritic integration of CA3 pyramidal neurons, we used two-photon glutamate uncaging at multiple synapses on thin dendritic segments in the basal (stratum oriens) and proximal apical (stratum radiatum) arborization (synapses here are primarily CA3 associational/recurrent; Amaral and Witter, 1989; Li et al., 1994) and compared the peak amplitude of the measured voltage responses to the arithmetic sum of the individual inputs (expected amplitude; Losonczy and Magee, 2006). Stimulating an increasing number of synapses with a high degree of synchrony (i.e., up to 20–40 inputs within a maximum of 6–12 ms, see Experimental Procedures and Supplemental Experimental Procedures available online) evoked depolarizations that were much larger than the expected sum in the vast majority of the dendrites (Figure 1). The deviation from linear integration progressively increased as input numbers were increased and reached a steady-state level (nonlinearity: 5.35 ± 0.43 mV additional depolarization, ∼50%–100% increase; n = 57 dendrites in 42 cells; Figures 1D and 1E) at a similar input range in all neurons (5–8 mV expected amplitude). The additional depolarization produced by nonlinear dendritic integration was largest in the apical dendrites (apical: 8.16 ± 1.45 mV, n = 9; basal: 4.83 ± 0.40 mV, n = 48; p < 0.05, Mann-Whitney test). The majority of the supralinear voltage response was generated by a slow component, although in many basal dendrites small amplitude fast spikelets were also observed (Figure 1B).

### Fast Dendritic Na^+^ Spikes Play a Relatively Minor Role

**Figure 2:**
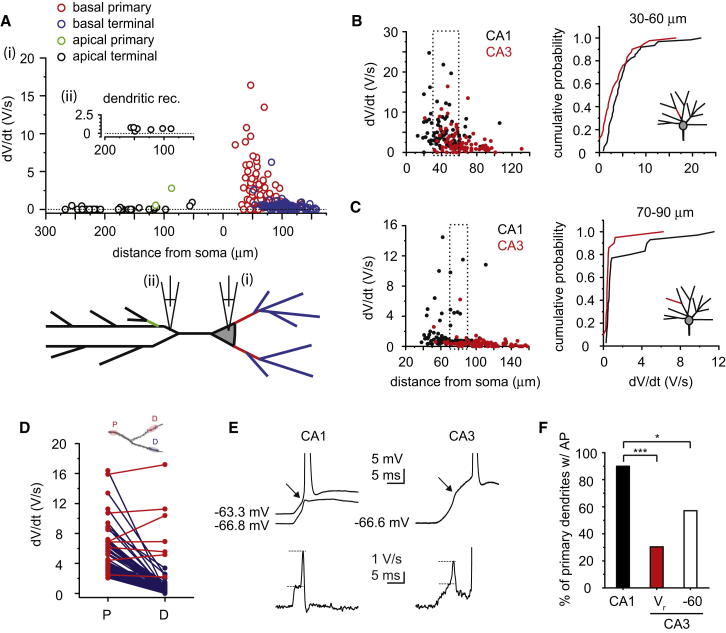
Properties of Fast Na^+^ Spikes (A) Na^+^ spike dV/dt versus distance of input site from the soma for basal primary (red dots) and terminal (blue dots) as well as apical primary (green dots) and terminal (black dots) branches. Input distance along oblique dendrites from apical trunks was 38 ± 3 μm (range: 12–69 μm; n = 34). (B and C) Left: distance-dependent distribution of dendritic Na^+^ spike strength in primary parent dendrites (B) and in terminal daughter dendrites (C) of CA1 (black, basal and apical perisomatic branches) and CA3 (red, basal branches) neurons. Right: cumulative probability of Na^+^ spike strength in the distance range indicated by the dashed box in the left panel (B, CA3: n = 41, CA1: n = 62, input 30–60 μm from soma, p < 0.05; C, CA3: n = 21, CA1: n = 30, input at 15–43 μm from the last branch point and 70–90 μm from soma, p < 0.01, Kolmogorov-Smirnov test). (D) Spike dV/dt of strong parent dendrites (P) and of connected daughter dendrites (D) in CA3 pyramidal cells. Red lines, functionally coupled P-D pairs (characterized by similar dV/dt values); blue lines, uncoupled pairs. Top schematic shows approximate stimulation positions. (E) Effect of Na^+^ spikes, evoked in perisomatic primary parent dendrites, on the AP output in CA1 and CA3 pyramidal cells. Top traces: Na^+^ spike evoked by synchronous uncaging (marked by arrow) directly evokes APs from V_rest_ in CA1 (left) but not in CA3 (right) pyramidal neurons. Hyperpolarization in CA1 reveals the strong Na^+^ spike triggering the precisely timed AP. Bottom: corresponding dV/dt traces. (F) Percentage of perisomatic primary parent dendrites in CA1 (n = 18/20) versus CA3 cells (at rest (V_r_), n = 27/89, p < 0.001, χ^2^ test; and at −60 mV, n = 8/14, p < 0.05, Fisher’s exact test) where the local Na^+^ spike directly evoked APs. Na^+^ spikes triggered in apical oblique dendrites were never strong enough to evoke an AP output (0/17 dendrites). Data are represented as mean ± SEM. See also Figure S1.

To dissect the underlying mechanism of the nonlinear component of dendritic integration, we first characterized the fast spikelet that was characteristic of a local Na^+^ spike (Ariav et al., 2003; Losonczy and Magee, 2006; Losonczy et al., 2008; Kim et al., 2012). The fast spikelet was indeed eliminated by 0.5–1 μM tetrodotoxin (TTX; n = 7; Figure S1A), confirming that it was mediated by voltage-gated Na^+^ channels (VGSCs). Determination of the somatic strength of dendritic Na^+^ spikes (by the rate of rise; dV/dt) generated in various regions of the arbor revealed a striking difference between apical and basal oblique dendrites (Figure 2A). While a fast spikelet could be observed in most basal dendrites (84.8%), similar stimulation generated Na^+^ spikes that were detectable at the soma in only a minority (29.4%) of apical dendrites, with a small average strength (0.65 ± 0.25 V/s, n = 10). Using apical trunk patch recordings (68 ± 9 μm from the soma, n = 3 recordings) and uncaging at synapses on nearby apical oblique dendrites (n = 7, branchpoint from patch pipette: 16 ± 9 μm, input site from branchpoint: 45 ± 7 μm, input site from soma: 130 ± 10 μm), we found that weak Na^+^ spikes were generated in all apical oblique branches studied (dV/dt = 0.59 ± 0.07 V/s, n = 7; Figure 2Aii), suggesting that poor spike propagation and dendritic filtering render most apical terminal branch dendritic Na^+^ spikes virtually undetectable at the soma. We also observed a tendency for subregion-dependent differences such that Na^+^ spikes in basal dendrites appeared to be stronger in pyramidal neurons from the CA3a subregion than in cells from CA3b and CA3c (Figures S1E and S1F).

Local Na^+^ spikes have been shown to play an important role in dendritic processing and storage in CA1PCs. To determine whether the Na^+^ spikes present in perisomatic dendrites of CA3PCs may fulfill a similar role, we systematically compared the properties of Na^+^ spikes in perisomatic dendrites of CA1 and CA3 pyramidal neurons. We found that Na^+^ spike threshold and overall hierarchic morphological distribution within the various dendritic compartments of the basal arbor (most CA3PCs lack proximal apical obliques in stratum lucidum) were similar to CA1 pyramidal neurons (Figures S1B–S1D). However, all measures of dendritic branch Na^+^ spike strength at the soma (Figures 2B–2D and Figure S1), including functional coupling (Figure 2D and Figures S1G–S1J) and action potential (AP) output generation (Figures 2E and 2F and Figure S1K) were weaker in CA3 than in CA1 regardless of the dendritic compartment in which they were evoked (see also Losonczy et al., 2008; Makara et al., 2009). In summary, Na^+^ spikes in almost all apical and most basal thin dendrites of CA3PCs have a relatively minor impact on AP output initiation or precision.

### Supralinear Integration Is Mainly Mediated by NMDA Receptors

**Figure 3:**
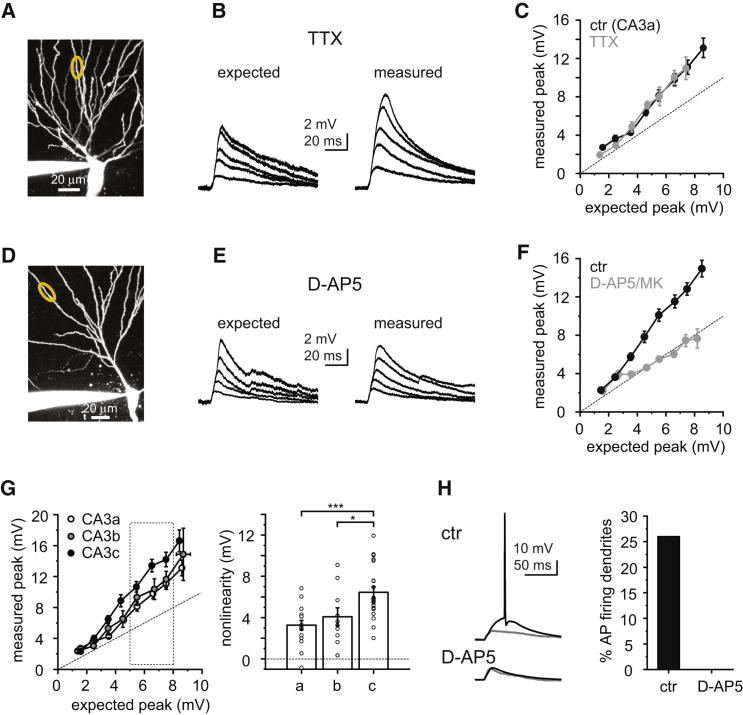
NMDARs Mediate Nonlinear Dendritic Integration in CA3 Pyramidal Cells (A) Stack two-photon image of a CA3 pyramidal cell. Yellow circle highlights stimulated dendritic segment. (B) Arithmetic sum (expected, left) and measured voltage responses evoked by uncaging at a variable number of synaptic inputs on the dendrite shown in (A) in the presence of 1 μM TTX. (C) Summarized effect of 0.5–1 μM TTX on the input-output function in CA3a pyramidal cells. Control data is also included in the data set of Figure 1D. Error bars for the x axis are within the symbols. (D) Stack two-photon image of a CA3 pyramidal cell. (E) Arithmetic sum (expected, left) and measured voltage responses evoked by synaptic inputs on the dendrite shown in (D) in the presence of 50 μM D-AP5. (F) Summarized effect of NMDAR blockers (50 μM D-AP5 or 20 μM MK801, pooled) on the input-output function. Control group is also included in the data set of Figure 1D. (G) Input-output function (left) and magnitude of nonlinearity (right) in the three CA3 subregions. Most x axis error bars are within the symbols. (H) Left: expected (gray) and measured (black) responses to synchronous synaptic stimulation in a control cell (top traces, 35 inputs) and in a different cell in the presence of 50 μM D-AP5 (bottom traces, 28 inputs). Cells were kept at V_rest_ (−68 to −72 mV). Right: percentage of the dendrites where APs were generated by synaptic stimulation in the 5–8 mV expected amplitude range. Data are represented as mean ± SEM. See also Figure S2.

In line with a lesser contribution of fast Na^+^ spikes, blockade of VGSCs with 0.5–1 μM TTX did not significantly affect dendritic input-output function even in basal dendrites of CA3a neurons where Na^+^ spikes were the strongest (nonlinearity in control: 3.26 ± 0.45 mV, n = 17; in TTX: 3.32 ± 0.66 mV, n = 7, p = 0.924, Mann-Whitney test; Figures 3A–3C) (Lavzin et al., 2012). In contrast, most of the nonlinear integration was produced by the slow component, suggesting that NMDARs were involved. Indeed, the NMDAR inhibitors D-AP5 (50 μM) or MK801 (20 μM) completely abolished the nonlinearity, turning integration into a linear form (Figures 3D–3F, nonlinearity in control: 5.12 ± 0.49 mV, n = 34; in D-AP5/MK801 pooled: 0.17 ± 0.38 mV, n = 12, p < 0.001, Mann-Whitney test). NMDA spikes were larger in the CA3c area than in CA3a and CA3b (Figure 3G, post hoc comparisons of mean ranks after Kruskal Wallis test, p < 0.001). This trend is the opposite of what was observed in the case of fast Na^+^ spikes (see Figures S1E and S1F), indicating a possible fine-tuning of dendritic integration mechanisms along the proximal-distal axis of CA3. Similar to the above results (and to cortical pyramidal neurons; Schiller et al., 2000; Polsky et al., 2009; Lavzin et al., 2012), focal electrical synaptic stimulation at selected basal dendritic segments (n = 7, distance from soma: 141 ± 15 μm) revealed a slow depolarizing component mediated by NMDARs (Figure S2), verifying NMDAR-mediated amplification (see Figure S2 for discussion).

The NMDA spike strongly contributed to suprathreshold depolarization at the soma (Figure 3H). With the membrane potential set close to the average V_rest_ (−68 to −72 mV), synchronous stimulation with expected amplitudes between 5 and 8 mV (heavily subthreshold for AP output) was able to evoke APs in 26.7% of dendrites (8 of 30 dendrites in 23 neurons; nonlinearity for suprathreshold: 7.68 ± 1.12 mV). In contrast, in the presence of D-AP5, stimulation in the same expected response range was never suprathreshold (0 of 9 dendrites in 6 neurons).

### Spatial and Temporal Integration by NMDA Spikes

**Figure 4:**
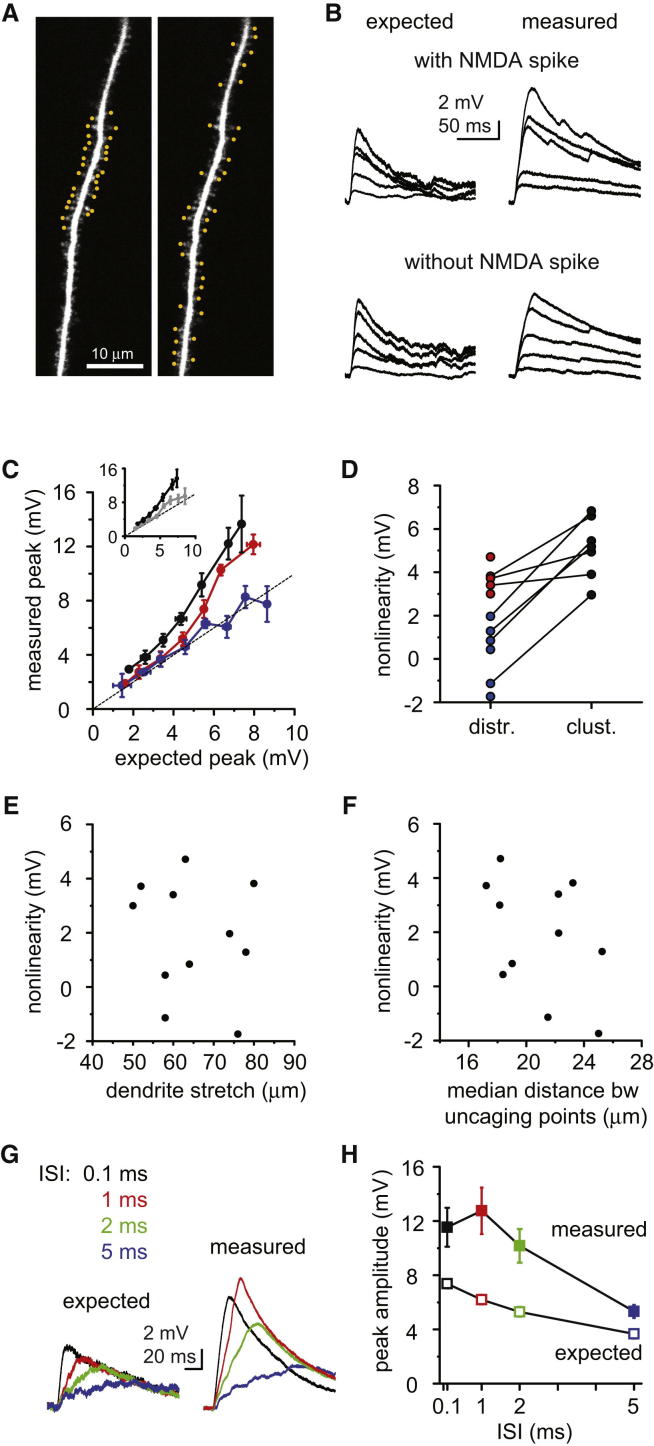
Spatial and Temporal Determinants of NMDAR-Mediated Nonlinear Integration (A) Stack two-photon image of a dendritic segment in the basal arbor, with synaptic inputs clustered in the middle (left) or distributed along the dendrite (right). (B) Representative examples with expected (left) and measured (right) voltage responses to a distributed input pattern in a dendrite integrating them in a nonlinear fashion (top traces, data from dendrite in A) and in another dendrite integrating them linearly (bottom traces). Responses to clustered stimulus are not shown. (C) Summary of the input-output relationship to distributed input. Blue, dendrites with linear integration (n = 6 out of 11 dendrites); red, dendrites with nonlinear integration (n = 5/11). Black, clustered input tested in some of the dendrites (n = 7). Inset: all experiments with distributed input pooled (gray, n = 11) versus clustered data (black, n = 7). (D) Comparison of nonlinearity to distributed versus clustered input. Color code is as in (C). (E and F) Relationship between the magnitude of nonlinearity evoked by distributed input and the length of the dendritic segment where the inputs were distributed (E) and the median distance between all uncaging points (F). (G) Representative experiment using different ISIs at 25 spatially clustered inputs. (H) Summary of the sensitivity of nonlinearity to input timing. Data are represented as mean ± SEM.

We next investigated the spatial and temporal integration window of NMDAR-mediated amplification. Inputs distributed on a longer stretch of dendrite (65 ± 3 μm, range: 50–80 μm, n = 11) could still evoke an NMDAR-mediated nonlinearity (>2 mV, as defined in Figure 1E) in about half of the dendrites (n = 5 out of 11 dendrites, nonlinearity: 3.73 ± 0.28 mV), whereas integration was linear in the remaining (n = 6 out of 11 dendrites, nonlinearity: 0.27 ± 0.58 mV, Figures 4A–4C). The nonlinearity generated with distributed input did not correlate significantly with the length of the stimulated dendritic segment (Figure 4E, Spearman R = −0.022, p = 0.946), nor with the distance between consecutive (data not shown) or all inputs (Figure 4F, Spearman R = −0.336, p = 0.313) in our limited data set. Clustered input in the middle of the same segment elicited NMDAR-mediated nonlinearity in all cases tested (three and four dendrites with and without nonlinear integration of distributed inputs, respectively, nonlinearity: 6.26 ± 0.66 mV, n = 7, Figures 4C and 4D). Thus, although the level of input clustering observed in other studies (Kleindienst et al., 2011; Takahashi et al., 2012) would be most efficient at producing NMDAR-dependent supralinear integration, such extreme clustering is not necessarily required.

Next, we studied the sensitivity of the NMDAR-mediated nonlinearity to input synchrony by stimulating 20–32 spatially clustered spines with different intervals between inputs (interstimulus interval [ISI], 0.1–5 ms). In contrast to Na^+^ spikes (Losonczy and Magee, 2006), NMDA spikes were reliably activated with 1–2 ms ISI, while more asynchronous input (ISI = 5 ms) generated a much smaller nonlinear component (Figures 4G and 4H, n = 9–10 for each ISI from n = 10 dendrites, p < 0.05, repeated-measures ANOVA; p < 0.05 for comparison of maximal supralinearity with ISI = 0.1–2 ms versus ISI = 5 ms, Wilcoxon test). Thus, the temporal requirements for NMDA spike generation are relatively loose. Based on these spatial and temporal integration rules, we speculate that NMDAR-mediated nonlinearity may play a role in dendritic integration of synaptic input patterns evolving over many tens of milliseconds during exploratory behavior.

### Bimodal Distribution of NMDA Spike Decay Time Course

**Figure 5:**
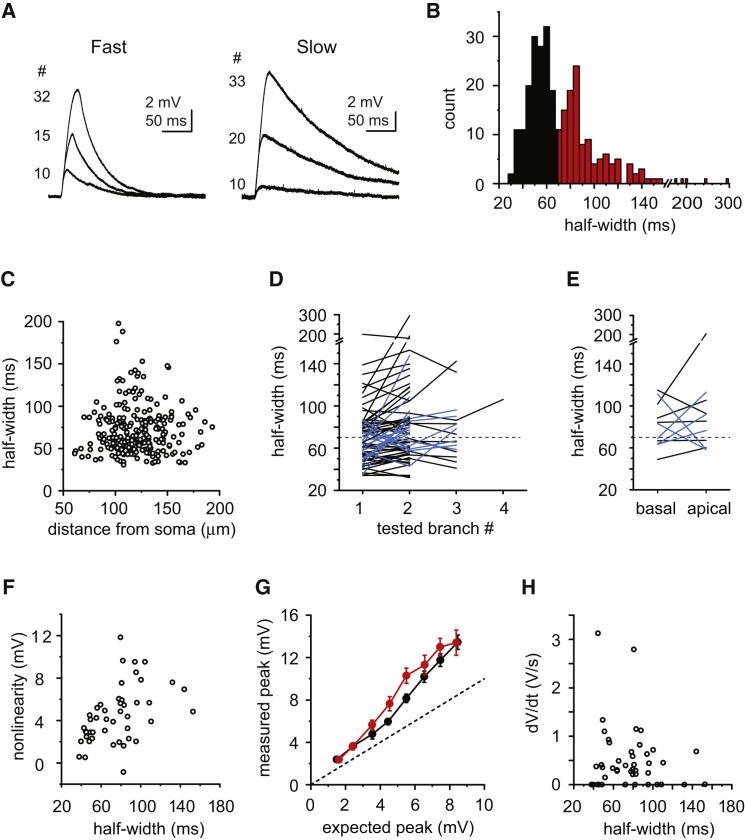
Variability of NMDA Spike Kinetics (A) Representative traces of NMDA spikes from two dendrites in different neurons, one with fast decay (left) and one with slow decay (right). (B) Histogram of the half-width (n = 280 dendrites in 182 cells). Black, fast spikes; red, slow spikes, separated at 70 ms. (C) Half-width versus distance from soma in basal dendrites (n = 258). (D) Half-width of NMDA spikes evoked in consecutively tested dendrites within the apical or in the basal arbor of individual cells. Black lines represent only fast or only slow dendrite groups (67 cells); blue lines represent mixed dendrite groups (18 cells). (E) As in (D) but for dendrites stimulated in the apical versus basal arborization of individual cells (mixed dendrites in 5/11 cells). (F) Relationship between half-width and the magnitude of nonlinearity (n = 46, basal dendrites). (G) Input-output relationship for fast (black, n = 19) and slow (red, n = 27) NMDA spikes. Data are represented as mean ± SEM. Error bars for the x axis are within the symbols. (H) Relationship between half-width and the strength of the fast Na^+^ spike in the same dendritic segment (n = 46, basal dendrites). See also Figure S3.

Analyzing the kinetics of voltage responses evoked by synchronous stimulation, we discovered that the decay (quantified as the half-width) showed substantial variability across dendrites. The distribution of the half-width differed from a normal distribution (p < 0.001, n = 280 dendrites, 258 basal, 22 apical, Shapiro-Wilks test) and rather formed a bimodal distribution with a group of NMDA spikes exhibiting fast decay and another population that decayed more slowly (peaks at ∼55 and ∼85 ms, respectively; Figures 5A and 5B). Accordingly, in all further analysis and experiments, we defined fast spikes as those with half-width <70 ms and slow spikes as those with half-width >70 ms. The kinetics of the NMDA spike in a given dendrite was relatively uniform over a range of peak amplitudes (Figure S3A). The half-width was not dependent on the particular synapses that were stimulated (Figures S3B and S3C). The half-width in basal dendrites was also not related to distance of the input site from the soma (Figure 5C, Spearman R = 0.032, p > 0.05) or morphological position in the branching arbor (Figure S3D). Somatic holding membrane potential was similar between the groups (V_m_, fast: −71.4 ± 0.4 mV, n = 19; slow: −72.2 ± 0.2 mV, n = 27, p = 0.063, Mann-Whitney test).

Fast and slow NMDA spikes in different branches of the same cell could be observed (Figures 5D, 5E, and S3E), especially when comparing apical and basal branches (Figures 5E and S3E), indicating some degree of dendritic compartmentalization of the underlying mechanism. The half-width of somatic APs was not different between cells where most dendrites expressed fast NMDA spikes versus those expressing mostly slow spikes (Figure S3F), suggesting that dendritic properties are responsible for the variable decay. We found a significant correlation between the half-width and the magnitude of nonlinearity of the NMDA spike (Figure 5F, Spearman R = 0.514, p < 0.05), and the input-output relationship was slightly shifted to the left in dendrites with slow NMDA spikes compared to that of fast NMDA spikes (Figure 5G, n = 19/27 fast/slow dendrites, p < 0.05 at 4–6 mV expected amplitude, Mann-Whitney test). On the other hand, NMDA spike half-width did not correlate with the strength of the Na^+^ spike evoked in the same dendrite (basal dendrites, Spearman R = 0.062, p = 0.680, Figure 5H).

### Channels Determining NMDA Spike Decay Time Course

Observations that the decay of the NMDA spike was voltage dependent (Figure S3G) and correlated with the somatic membrane time constant (n = 18, Spearman R = 0.760, p < 0.05, Figure S3H) suggested that an active voltage-dependent conductance is regulating the decay of NMDA spikes. Several K^+^ channel types have been described in CA3PCs (Storm, 1990). We aimed to dissect the role of K^+^ currents in regulating dendritic spikes using various K^+^ channel modulators.

**Figure 6:**
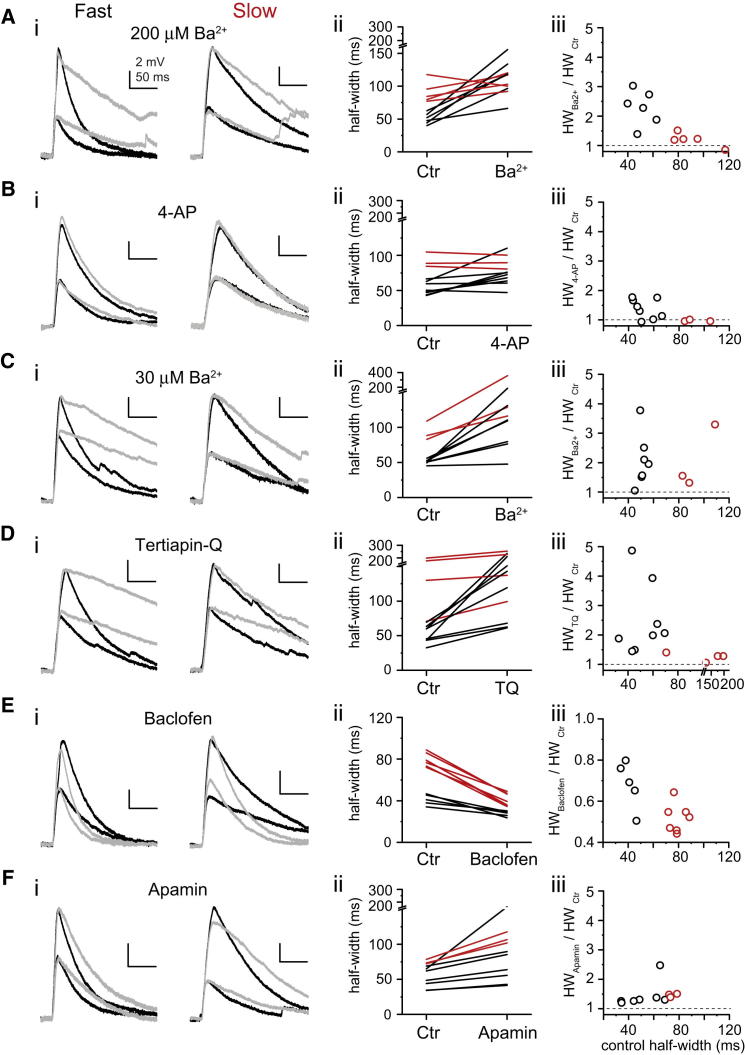
Regulation of NMDA Spike Decay by K^+^ Currents (A–F) (i) Representative individual traces at two stimulation strengths (different laser power or synapse number) in dendrites with fast (left) or slow (right) spikes before (black) and after (gray) bath application of various K^+^ current modulators. Traces with similar amplitudes (not necessarily same laser power) are displayed for clear comparison of decay kinetics. (A) Ba^2+^ (200 μM); (B) 4-AP (2 mM, in the presence of 1 μM TTX); (C) Ba^2+^ (30 μM); (D) tertiapin-Q (0.5 μM); (E) baclofen (20 μM); (F) apamin (0.1 μM). (ii) Summary of the effect of K^+^ current modulators on NMDA spike half-width (black, half-width < 70 ms, red: half-width > 70 ms). (iii) Fractional effect of K^+^ current modulators on NMDA spike half-width (black, half-width < 70 ms, red: half-width > 70 ms). See also Figure S4.

To narrow down the pool of candidates, we first applied a moderately low concentration of Ba^2+^ (200–250 μM, for 10 min in the bath but not in the puffer pipette) that should have no significant effect on delayed rectifier, Ca^2+^-activated and transient D-type K^+^ currents, or the hyperpolarization-activated cation channel I_h_ but blocks inward rectifier K^+^ (IRK) currents and partially inhibits transient A-type (Gasparini et al., 2007; Losonczy et al., 2008) and certain “leak” K^+^ currents (Coetzee et al., 1999). Fast NMDA spikes were unanimously prolonged by 200–250 μM Ba^2+^, rendering them slow (half-width ctr: 50.5 ± 3.5 ms, Ba^2+^: 114.7 ± 12.6 ms, n = 6, p < 0.05, Wilcoxon test, Figure 6A, see also Figures S4A and S4B for effect of K^+^ channel modulators on V_m_ and input resistance), whereas the kinetics of slow NMDA spikes was not affected by the same treatment (half-width ctr: 90.8 ± 7.4 ms, Ba^2+^: 106.4 ± 5.3 ms, n = 5, p = 0.138, Wilcoxon test). As a time control, repeated activation of NMDA spikes in 10 min induced only a slight, nonsignificant increase in the half-width of fast NMDA spikes (half-width ctr: 47.6 ± 4.4 ms, after 10 min: 55.0 ± 5.7 ms, n = 8, p = 0.092, Wilcoxon test).

Transient A-type K^+^ currents regulate dendritic Na^+^ spikes and are partially inhibited by 200–250 μM Ba^2+^ in CA1PCs (Gasparini et al., 2007; Losonczy et al., 2008). To more specifically test the involvement of A-type current in the regulation of NMDA spike decay in CA3PCs, we applied 4-aminopyridine (2 mM 4-AP +1 μM TTX to control network activity, see Experimental Procedures). 4-AP applied in the presence of TTX prolonged the half-width of fast NMDA spikes (TTX only: 52.7 ± 3.2 ms, TTX + 4-AP: 71.7 ± 6.5 ms, n = 8, p < 0.05, Wilcoxon test, Figures 6B and S4), but the effect was nearly half that of 200–250 μM Ba^2+^ (fractional change: 4-AP: 1.37 ± 0.12; 200–250 μM Ba^2+^: 2.29 ± 0.24, p < 0.05, Mann-Whitney tests with Bonferroni correction for fractional change comparisons after Kruskal-Wallis test, p < 0.01), indicating that transient voltage-gated K^+^ currents play only a partial role in the regulation of this parameter. This result is consistent with the notion that the majority of transient K^+^ channels are expected to inactivate during the slow time course of NMDA spikes.

Among K^+^ currents, inward rectifiers (IRKs) have the highest sensitivity to external Ba^2+^ (Hibino et al., 2010). We next tested a lower concentration of Ba^2+^ (30 μM), which should completely block IRK channels, while having only a minimal effect on other types of K^+^ currents. We found that 30 μM Ba^2+^ reversibly increased the half-width of fast NMDA spikes to a similar degree as that measured with 200–250 μM Ba^2+^ (Figures 6C and S4, control: 51.1 ± 1.3 ms, Ba^2+^: 106.0 ± 17.0 ms, n = 7, p < 0.05, Wilcoxon test; comparison of fractional change with 200–250 μM Ba^2+^: p = 0.520), indicating that an IRK current plays a dominant role in determining the decay characteristics of NMDA spikes.

G-protein-activated inward rectifier K^+^ (GIRK) channels (Lüscher and Slesinger, 2010) are abundant in dendrites and spines of CA3PCs where they are found in tight association with GABA_B_ receptors (GABA_B_Rs) (Gähwiler and Brown, 1985; Sodickson and Bean, 1996; Lüscher et al., 1997; Koyrakh et al., 2005; Kulik et al., 2006). The GIRK channel inhibitor tertiapin-Q (0.5 μM; Jin and Lu, 1999) prolonged the half-width of fast NMDA spikes to a similar degree as Ba^2+^ (Figures 6D and S4, control: 52.2 ± 4.5 ms, tertiapin-Q: 131.2 ± 23.6 ms, n = 8, p < 0.05, Wilcoxon test; fractional change comparison with Ba^2+^: tertiapin-Q: 2.50 ± 0.44, n = 8, Ba^2+^ [30–250 μM pooled]: 2.17 ± 0.21, n = 13, p = 0.856), while affecting slow NMDA spikes relatively weakly (control: 143.7 ± 28.1 ms, tertiapin-Q: 179.0 ± 36.4 ms, n = 4, p = 0.125, Wilcoxon test). On the contrary, increasing GIRK channel activity via GABA_B_R stimulation (20 μM baclofen) strongly reduced the half-width of slow NMDA spikes (Figures 6E and S4, control: 79.1 ± 2.4 ms, baclofen: 41.0 ± 2.4 ms, n = 7, p < 0.05, Wilcoxon test), while having less effect on fast NMDA spikes (control, 41.0 ± 2.3 ms, baclofen, 27.5 ± 1.3 ms, n = 5, p < 0.05, Wilcoxon test). Focal dendritic application of baclofen induced somatic hyperpolarization and inwardly rectifying K^+^ current, confirming robust, though variable, expression of functional GIRK channels in CA3PCs (Figures S4E–S4J). These results altogether strongly implicate GIRK channels to be the primary determinant of NMDA spike decay.

Because NMDARs induce large local Ca^2+^ signals and have been shown to be functionally coupled to small conductance Ca^2+^-activated (SK) K^+^ channels in spines (Ngo-Anh et al., 2005), we next examined the role of SK channels in the regulation of NMDA spike decay. The SK channel blocker apamin (0.1 μM) mildly but significantly increased the half-width of fast NMDA spikes (Figures 6F and S4, control: 51.0 ± 5.5 ms, apamin: 77.1 ± 15.7 ms, n = 7, p < 0.05, Wilcoxon test). The effect of apamin appeared to be similar in dendrites regardless of the initial NMDA spike half-width indicating that fast and slow spikes were uniformly regulated by SK. In contrast, inhibition of large conductance Ca^2+^-activated K^+^ channels by iberiotoxin (0.1 μM) had no significant effect on half-width of fast NMDA spikes (Figure S4D, control: 47.3 ± 3.5 ms, iberiotoxin: 54.9 ± 6.4 ms, n = 6, p = 0.115, Wilcoxon test).

In summary, the above results strongly indicate that variable activity of GIRK currents dominantly regulates the time course of large voltage responses evoked by correlated synaptic activity in CA3PCs, with a lesser contribution by SK and A-type K^+^ currents.

### NMDA Spike Decay Determines AP Output

**Figure 7:**
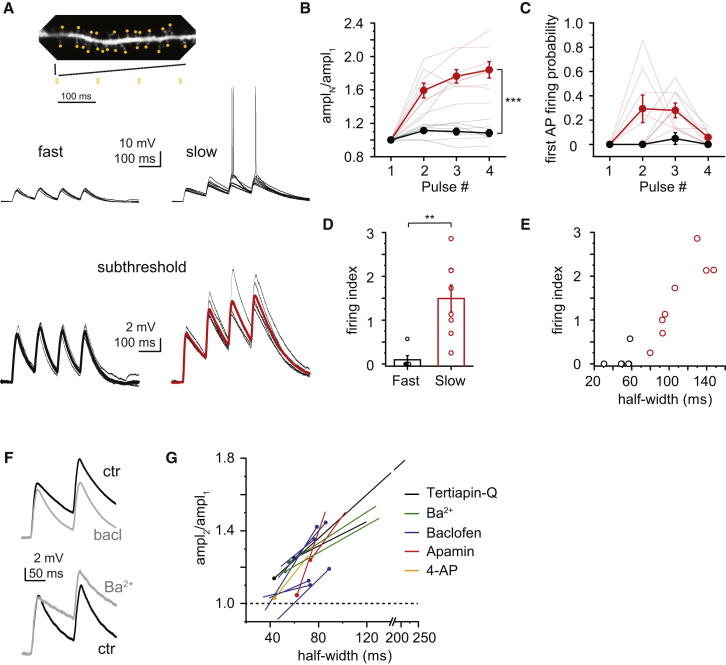
NMDA Spike Decay Determines AP Output to Theta-Modulated Repetitive Synaptic Input (A) Top: theta stimulus paradigm. Middle: representative responses from dendrites with fast (left) and slow (right) NMDA spikes. Bottom: magnified traces from middle panel subthreshold to AP generation. Thick traces represent average. (B) Summary of summation by theta paradigm of fast (black) and slow (red) NMDA spikes. (C) Probability of generating the first AP among the four theta stimulation cycles in fast (black) and slow (red) NMDA spikes. (D) Firing index (see Experimental Procedures) in the two groups. (E) Relationship between half-width and firing index. The longer the half-width, the earlier the first AP-generating stimulation cycle was. (F) Representative recordings (average of at least three traces) demonstrating the effect of baclofen (top) and 30 μM Ba^2+^ (bottom) on paired-pulse summation of slow and fast NMDA spikes, respectively. (G) Summary of the effect of various K^+^ current modulators on the half-width and paired-pulse summation (20–25 inputs, 100 ms delay). Each line represents an individual experiment in which substantial change in half-width occurred. Dotted end on each line marks control condition. Data are represented as mean ± SEM. See also Figure S5.

What is the impact of NMDA spike decay on the physiological response of CA3 pyramidal neurons? During in vivo exploratory behavior, basic units of place-coding neuronal ensembles are thought to fire repeatedly at consecutive cycles of the theta rhythm, constrained by gamma oscillation cycles (Buzsáki, 2010). Thus, when the animal enters the place field of an ensemble, the ensemble members receive input from their partners approximately every ∼100–200 ms. Since this interval falls in the range of the NMDA spike duration, the decay of the voltage response could determine the (local or global) temporal summation of such repeated input. To test this hypothesis, we stimulated synchronous synaptic inputs (25 clustered spines with 0.1 ms interspine intervals) four times with 100 ms delays between stimulations (theta protocol; 9.3 Hz). Experimental conditions were set so that V_m_ and the amplitude of the first NMDA spike were indistinguishable between fast- and slow-spiking dendrites (V_m_, fast: −70.4 ± 0.3 mV, n = 7, slow: −70.0 ± 0.1 mV, n = 10, p = 0.407, Mann-Whitney test; first response peak amplitude, fast: 8.27 ± 0.16 mV, n = 7, slow: 8.20 ± 0.15 mV, n = 10, p = 0.696, Mann-Whitney test). Using the theta protocol, we found a dramatic difference in summation and AP output between fast and slow NMDA spikes. While peak depolarization by fast NMDA spikes did not increase significantly even by the fourth stimulation (fourth/first amplitude = 1.08 ± 0.04, n = 7, p = 0.090, Wilcoxon test), slow NMDA spikes summed efficiently, generating 1.84 ± 0.09 times larger amplitude by the fourth stimulation (n = 8, repeated-measures ANOVA: interaction between half-width and summation, p < 0.001, Figures 7A and 7B). Analysis of summation of two stimulations in a larger data set showed a strong correlation between summation and half-width (n = 25, Spearman R = 0.895, p < 0.05, Figure S5). As a result, the AP output achieved by theta stimulation of fast and slow NMDA spikes was also dramatically different. Theta stimulation of fast NMDA spikes evoked APs in only one out of six cases (six dendrites in five cells, Figures 7A and 7C–7E). In contrast, theta stimulation of slow NMDA spikes triggered APs in every experiment (eight out of eight dendrites in five cells, p < 0.01, Fisher’s exact test, Figures 7A and 7C), usually by the second or third cycle (Figure 7C). The longer the half-width, the earlier stimulation cycle was successful in evoking APs (firing index, see Experimental Procedures, fast: 0.10 ± 0.10, n = 6, slow: 1.49 ± 0.31, n = 8, p < 0.01, Mann-Whitney test, Figures 7D and 7E). Finally, we tested the effect of various K^+^ current modulators to verify that manipulation of NMDA spike decay consequently leads to changes in summation. To avoid plasticity and photodamage issues, we limited our measurements to the degree of summation occurring in response to paired-pulse stimulation. The K^+^ current inhibitors Ba^2+^, tertiapin-Q, 4-AP, and apamin as well as the K^+^ current activator baclofen induced changes in paired-pulse summation proportional to their effect on NMDA spike half-width (Figures 7F and 7G). In summary, the reliability of AP output during theta stimulation is fundamentally determined by the decay of the NMDA nonlinearity that, in turn, is primarily regulated by GIRK channels with some additional contribution by SK and A-type K^+^ channels. This compartment-specific regulation of branch excitability would therefore allow input onto more excitable dendrites to preferentially drive AP output.

## Discussion

Dendritic processing of patterned synaptic input may support the generation and consolidation of information coding neuronal ensembles within the CA3 network. Here we show that dendritic integration of synchronous synaptic inputs is highly supralinear in thin dendrites of CA3 pyramidal neurons and this supralinearity is mainly dependent on NMDARs with some contribution by fast Na^+^ spikes. The decay of voltage responses generated by synchronous inputs is regulated by K^+^ (mainly GIRK) channel function, thereby allowing selective amplification of theta-modulated repetitive input patterns.

### Dendritic Integration in the Hippocampal Circuit

Recent studies investigating dendritic integration in CA1 pyramidal neurons and dentate gyrus granule cells revealed that these two types of hippocampal principal neurons express very different integrative properties. While the initiation of composite Na^+^ and NMDA spikes in CA1 pyramidal neuron dendrites enables them to produce strong supralinear integration (Ariav et al., 2003; Losonczy and Magee, 2006), granule cells integrate inputs in an essentially linear fashion (Krueppel et al., 2011). This suggests that specific forms of dendritic integration may support different computational capabilities. Linear integration may allow sparsification and orthogonalization in the dentate gyrus through a true winner-take-all selection mechanism, whereas fast Na^+^ spikes evoking precisely timed APs may promote synchronized output of CA1 cells coding the same complex input features during SWRs. Here we describe dendritic integration of synaptic inputs in principal neurons of CA3. Our results demonstrate that integrative properties of thin apical and basal dendrites of CA3PCs differ from the other two principal neuron types. They are obviously different from DG granule cells in that they express active integrative mechanisms that enable strongly supralinear integration of spatiotemporally correlated input patterns. Yet, unlike perisomatic dendrites of CA1PCs where dendritic Na^+^ spikes are remarkably strong even measured at the soma, the dominant form of dendritic supralinearity in CA3 pyramids is mediated by NMDARs, and the contribution of fast Na^+^ spikes to the somatic response is relatively minor in the vast majority of even basal dendrites. These properties resemble integration in thin dendrites of L2/3 and L5 cortical pyramidal neurons (Schiller and Schiller, 2001; Antic et al., 2010; Branco et al., 2010). NMDA spikes in CA3PCs provide robust amplification of the voltage response to synchronous input involving more than ∼15 synapses. Furthermore, NMDA spikes are less sensitive to input timing than Na^+^ spikes: ∼20 inputs with 2 ms intervals reliably generated supralinear integration, whereas similar temporal patterns failed to activate local Na^+^ spikes in CA1 neurons (Losonczy and Magee, 2006). Thus, CA3PC dendrites may efficiently amplify less coherent (but still coincident) synaptic inputs, such as those provided by activity of a memory-coding ensemble structured by theta-gamma oscillation during exploratory behavior. NMDA spikes have a special relationship with burst firing in that bursting input is a particularly effective stimulus and the spikes themselves enhance bursting output (Polsky et al., 2009). These properties fit with the well-known bursting properties of CA3PCs (Ranck, 1973; Buckmaster and Amaral, 2001; Mizuseki et al., 2012).

Our results are in accordance with the recent report of Kim et al. (2012) that demonstrated that thick CA3PC dendrites can actively generate local Na^+^ spikes upon dendritic current injection. In contrast to that study, we stimulated synaptic inputs by two-photon glutamate uncaging in thin dendrites of CA3PCs. While we also detected local dendritic Na^+^ spikes, we found that Na^+^ spikes were relatively weak as measured at the soma (especially in the apical arbor) and that supralinearity of integration was rather provided by NMDARs, a mechanism that could not be studied by the direct current injection used by Kim et al. (2012). Although favoring the initiation of dendritic spikes, the morphological structure of CA3PCs (frequent branching of the apical trunk to several thinner trunks) may lead to strong attenuation of fast Na^+^ spikes as they propagate to the soma, while slower NMDA spikes should be less affected.

### Regulation of NMDA Spike Decay by K^+^ Currents

Modifiable dendritic K^+^ currents have been widely implicated in the regulation of synaptic plasticity and dendritic function (Shah et al., 2010). The A-type K^+^ current received much interest for promoting localized alterations in dendritic function (Frick et al., 2004; Losonczy et al., 2008), while less attention has been focused on the role of other K^+^ channels. GIRK channels are activated by various G_i_-protein-coupled receptors (Lüscher and Slesinger, 2010), are abundant in dendrites and spines of CA3PCs in tight association with GABA_B_Rs (Gähwiler and Brown, 1985; Sodickson and Bean, 1996; Lüscher et al., 1997; Koyrakh et al., 2005; Kulik et al., 2006), and have been recorded in the apical trunk of CA1 and cortical pyramidal neurons (Chen and Johnston, 2005; Takigawa and Alzheimer, 1999; Breton and Stuart, 2012; Palmer et al., 2012). Consistent with the above data, we found robust, though variable, expression of functional GIRK channels in CA3PC basal distal dendrites. Due to the intrinsic voltage dependency of their conductance, dendritic IRK currents may be well positioned to favor nonlinear processing of spatiotemporally clustered synaptic inputs. High IRK conductance at V_rest_ reduces input resistance and the time and length constants, thereby limiting integration of distributed synaptic inputs. However, the arrival of sufficient excitatory synaptic input may diminish this restraining K^+^ conductance, giving place to effective spatiotemporal summation of EPSPs and nonlinear interactions via stronger activation of NMDARs (Wessel et al., 1999; Sanders et al., 2013). When NMDAR activation ceases, the GIRK conductance could contribute to the gradual restoration of the resting membrane potential, shaping the time course of repolarization.

Our results demonstrate variable regulation of dendritic NMDA spike decay even among different dendrites of individual CA3PCs, a regulation that appears to be mainly mediated by the variable activity of GIRK channels. Because the function of GIRK channels depends on several factors such as the density and background activity of the channels themselves, the constitutive activity of metabotropic receptors or the ambient concentration of their extracellular agonists, further experiments are needed to explore the mechanism behind this inhomogeneity. However, it is tempting to hypothesize that it may be a fingerprint of the in vivo history of synaptic activity inducing experience-related long-term plasticity (downregulation) of GIRK channel function, leading to increased excitability in the affected dendritic regions. Such plasticity could be mechanistically reminiscent of the experience-related long-term plasticity (BSP) of Na^+^ spike propagation in CA1PCs, which is mediated by compartmentalized downregulation of transient K^+^ currents (Losonczy et al., 2008; Makara et al., 2009). However, in contrast to BSP, which primarily regulates the timing and precision of AP output, this novel form of plasticity would rather affect the reliability (and not the timing) of the AP output via modulation of GIRK channels. Beside such a potential long-term plasticity, GIRK activity can be also adjusted on short term by various neurotransmitters and neuromodulators (e.g., GABA), providing a mechanism for fine-tuning of dendritic integrative properties on different timescales.

### Functional Implications of NMDA Spikes with Variable Decay

NMDAR-mediated dendritic amplification and its regulation by GIRK channels could well support memory storage functions of the autoassociative CA3 network. An important feature of such a system is its ability for pattern completion (Marr, 1971; Rolls and Kesner, 2006; Nakazawa et al., 2002; Guzowski et al., 2004; Lee et al., 2004; Gold and Kesner, 2005). Pattern completion requires that the interaction between ensemble members becomes strong enough to provide suprathreshold depolarization even by degraded input patterns. While synaptic plasticity is generally thought to be the main cellular mechanism involved (Marr, 1971; McNaughton and Morris, 1987; Treves and Rolls, 1994; Kleindienst et al., 2011), NMDA spikes evoked by spatiotemporally correlated synaptic activity would further promote reliable firing and could also be involved in the induction of synaptic plasticity. This mechanism could not only act as a dendritic gain but also enhance the discriminative power of the network by ensuring reliable firing specifically of those neurons that receive a sufficient number of synchronous and repetitive inputs (Legenstein and Maass, 2011). Intriguingly, NMDARs in CA3 have been shown to be important for pattern completion (Nakazawa et al., 2002; Fellini et al., 2009; Kesner and Warthen, 2010). While this effect has been considered to implicate synaptic plasticity in the phenomenon, NMDAR-mediated dendritic integration could also be involved. Altogether, our results support the notion that regulation of dendritic integration in a cell-type-specific and compartmentalized manner provides a wide array of dynamic learning rules to promote complex computational functions of cortical networks.

## Experimental Procedures

### Hippocampal Slice Preparation and Patch-Clamp Recordings

Adult male Sprague-Dawley rats (8–12 weeks old) were used to prepare transverse slices (400 μm) from the hippocampus similarly to that described previously (Losonczy and Magee, 2006), according to methods approved by the Janelia Farm Institutional Animal Care and Use Committee and by the Animal Care and Use Committe (ACUC) of the Institute of Experimental Medicine, Hungarian Academy of Sciences, in accordance with DIRECTIVE 2010/63/EU Directives of the European Community and Hungarian regulations (40/2013, II.14.) (see Supplemental Experimental Procedures). Slices were incubated in a submerged holding chamber in artificial cerebrospinal fluid (ACSF) at 35°C for 30 min and then stored in the same chamber at room temperature. For recording, slices were transferred to a custom-made submerged recording chamber under the microscope where experiments were performed at 33°C–35°C in ACSF containing 125 mM NaCl, 3 mM KCl, 25 mM NaHCO_3_, 1.25 mM NaH_2_PO_4_, 1.3 mM CaCl_2_, 1 mM MgCl_2_, 25 mM glucose, 3 mM Na-pyruvate, and 1 mM ascorbic acid, saturated with 95% O_2_ and 5% CO_2_. In focal stimulation experiments, CaCl_2_ concentration was raised to 2 mM to facilitate release. Cells were visualized using an Olympus BX-61 or a Zeiss Axio Examiner epifluorescent microscope equipped with differential interference contrast optics under infrared illumination and a water-immersion lens (60×, Olympus, or 63×, Zeiss). Current-clamp whole-cell recordings from the somata of hippocampal CA3 (or in some experiments CA1) pyramidal neurons were performed using a BVC-700 amplifier (Dagan) in the active “bridge” mode, filtered at 3 kHz and digitized at 50 kHz (except for experiments in Figures S4E–S4G, where 10 kHz was used). Voltage-clamp experiments (Figures S4H–S4J) were performed with an Axopatch 200B amplifier (Molecular Devices), filtered at 2 kHz and digitized at 10 kHz. Patch pipettes (2–6 MΩ) were filled with a solution containing 120 mM K-gluconate, 20 mM KCl, 10 mM HEPES, 4 mM NaCl, 4 mM Mg_2_ATP, 0.3 mM Tris_2_GTP, 14 mM phosphocreatine (pH = 7.25), complemented with 50–100 μM Alexa Fluor 488 (for uncaging experiments; all fluorescent dyes from Invitrogen-Molecular Probes) or 100 μM Oregon green BAPTA-1 (OGB-1) and 50 μM Alexa Fluor 594 (for focal electrical stimulation experiments). In some experiments, the pipette solution included ∼0.1%–0.3% biocytin (Sigma). Alexa Fluor 488 or 594 fluorescence or biocytin labeling with immunoperoxidase reaction was used for post hoc verification of the localization of neurons along the proximodistal axis of CA3. Series resistance was <30 MΩ. CA3 neurons had resting membrane potentials between −60 and −76 mV (average: −68.0 ± 0.2 mV, n = 381). CA3PCs were usually kept at −68 to −72 mV, unless otherwise indicated. CA1 PCs were held at ∼−65 mV unless otherwise indicated.

### Two-Photon Imaging and Uncaging

A dual galvanometer-based two-photon scanning system (Prairie Technologies) was used to image Alexa Fluor 488-loaded neurons and to uncage glutamate at individual dendritic spines (Losonczy and Magee, 2006; Losonczy et al., 2008; Makara et al., 2009). Two ultrafast pulsed laser beams (Chameleon Ultra II; Coherent) were used, one at 920 nm for imaging Alexa Fluor 488 and the other at 720 nm to photolyze MNI-caged-L-glutamate (Tocris; 10 mM) that was applied through a puffer pipette with an ∼20- to 30-μm-diameter, downward-tilted aperture above the slice using a pneumatic ejection system (Picospritzer III [Parker Hannifin] or PDES-02TX [NPI]). Laser beam intensity was independently controlled with electro-optical modulators (Model 350-50, Conoptics).

Local dendritic spikes were evoked by uncaging of MNI-glutamate at a spatially clustered set of visually identified spines (see also Supplemental Experimental Procedures) with the highest synchrony our system allows, using 0.2 ms uncaging duration with 0.1 ms intervals between synapses (termed synchronous stimulation), unless otherwise indicated. For clarity, throughout the paper the term “Na^+^ spike” refers to local Na^+^ channel-mediated dendritic spikes, whereas the axosomatically generated spike is termed action potential or AP. Dendritic Na^+^ spikes were usually evoked using 5–20 synapses.

NMDAR-mediated nonlinearity was measured in dendritic segments 61–193 μm (basal dendrites) or 152–315 μm (apical dendrites) from the soma. NMDA spikes were generated using 20–40 synapses activated synchronously (0.2 ms uncaging duration and 0.1 ms intervals between synapses), except in some experiments (Figures 4G and 4H) in which longer intervals (1–5 ms) were used. To avoid variability in kinetic parameters of NMDA spikes due to distance-dependent distortion of voltage responses, we performed pharmacological experiments, spatiotemporal distribution experiments, and paired-pulse experiments exclusively on basal dendrites. For determining input-output function, the expected amplitude was calculated as the arithmetic sum of the physiologically sized unitary gluEPSPs at the given temporal input pattern. Unitary gluEPSPs were measured repeatedly (usually two to five times) interleaved with synchronous stimulations, using 205–420 ms intervals between the individual synapses (see also Supplemental Experimental Procedures). When necessary, the cell was hyperpolarized to avoid action potential firing in order to measure NMDA spike amplitude. Because of the voltage dependence of NMDA spike half-width (Figure S3G), V_m_ was kept between −68 to −74 mV (usually at −70 to −73 mV) in experiments involving half-width measurement. When comparing paired-pulse dynamics and AP coupling of fast and slow NMDA spikes (Figure 7), the holding V_m_ was set to ∼68.5–71.0 mV and only traces where amplitude of the first pulse response was between 7 and 10 mV were analyzed. Firing index was calculated as follows: each trace was assigned a score = 5-N, where N is the number of the pulse where the first AP occurred (i.e., if the AP occurred on cycle 3: N = 3, score = 2; if no AP occurred then N = 5 was used, resulting in score = 0), and the score of all traces was averaged.

The latency and jitter of APs evoked by dendritic spikes (Figure S1K) was determined in cells where several traces using the same laser power and synapse number were obtained. The average AP threshold, measured on the uncaging evoked slow component, was −52.2 ± 0.7 mV (n = 39 cells). The membrane time constant (Figure S3H) was measured as the slowest time constant of a multiexponential curve fitted on the average voltage response evoked by 20 pA, 300 ms hyperpolarizing step current injections. Input resistance (Figure S4B) was determined at the end of voltage responses to 50–100 pA, 300 ms hyperpolarizing step current injections. Where the propensity of Na^+^ or NMDA spikes to generate AP output was examined, all cases were considered positive when at least one stimulation trace evoked the AP.

D-AP5, MK801, TTX, tertiapin-Q, baclofen, apamin, and iberiotoxin (all from Tocris) were dissolved in distilled water in stock solutions; aliquots were stored at −20°C and used on the day of experiment. 4-AP (Tocris) was directly dissolved into the extracellular solution immediately before use (see also Supplemental Experimental Procedures). When used for input-output measurements (Figure 3), D-AP5, MK801, and TTX were usually present in the bath as well as in the puffer pipette, and drug-treated cells were compared to the control cell group. When testing their effect on NMDA spike half-width (Figures 6, 7F, and 7G), K^+^ channel modulators and TTX were applied in the bath only and statistical comparison was made between control and drug-treated conditions in the same cells. It should be noted that the effective concentration of drugs applied this way is somewhat reduced during puffing of drug-free MNI-glutamate solution for uncaging. For comparison of decay kinetics before and after drug application, the laser power was adjusted to yield voltage responses of 6–12 mV under both conditions. Because bath application of 4-AP induced epileptiform activity in the slice (data not shown), 4-AP experiments were performed in the presence of 1 μM TTX to silence network activity. TTX itself had no significant effect on NMDA spike half-width (control: 58.8 ± 7.0 ms, TTX: 65.5 ± 8.4 ms, n = 6, p = 0.248, Wilcoxon test). For details of focal dendritic baclofen application experiments (Figures S4E–S4J) and focal electrical stimulation experiments (Figure S2), see Supplemental Experimental Procedures.

### Data Analysis

Analysis was performed using custom-written macros in IgorPro (WaveMetrics). Detailed morphological and distance measurements were performed on stacked images of Alexa Fluor 488- or 594-loaded neurons (collected at the end of the experiment) using ImageJ (NIH). Distances were measured from the approximate midpoint of the input site. Statistical analysis was performed using Statistica software (Statsoft). N values represent number of dendrites unless otherwise indicated. Differences were considered significant when p < 0.05. In all figures, symbols and error bars represent mean ± SEM. ^∗^p < 0.05; ^∗∗^p < 0.01; ^∗∗∗^p < 0.001.

## Supplemental Information

Document S1. Supplemental Experimental Procedures and Figures S1–S5
