# Supplementary material for: Variable Dendritic Integration in Hippocampal CA3 Pyramidal Neurons
Source: Neuron. 2013 Dec 18;80(6):1438–50. doi: 10.1016/j.neuron.2013.10.033 (PMC3878388; doi:10.1016/j.neuron.2013.10.033)
Supplement: Document S1. Supplemental Experimental Procedures and Figures S1–S5 [file mmc1.pdf]

Neuron, Volume *80*

Supplemental Information

Variable Dendritic Integration in Hippocampal CA3 Pyramidal Neurons

Judit K. Makara and Jeffrey C. Magee

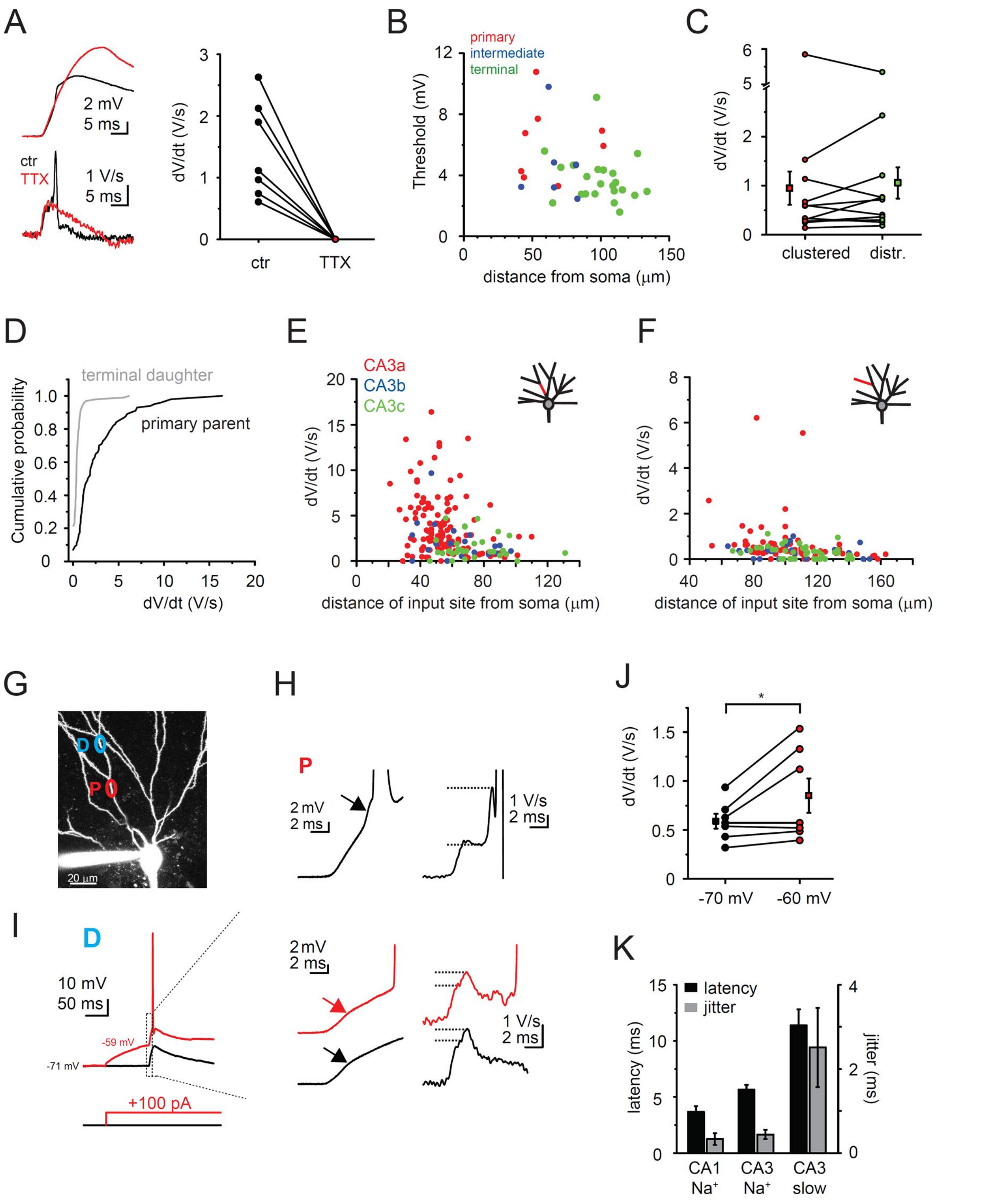

Figure S1. Properties of fast dendritic Na^+ spikes, related to Figure 2.
(A) Effect of 0.5 μM TTX on the fast spikelet. Left, representative voltage responses (upper traces) and corresponding dV/dt (lower traces) evoked by synchronous glutamate uncaging in a dendritic segment before (ctr, black) and after (TTX, red) application of TTX. Note that in TTX even stronger stimulation (generating larger EPSP) failed to evoke Na^+ spike. Right, dV/dt values

You created this PDF from an application that is not licensed to print to novaPDF printer (http://www.novapdf.com)

in individual experiments before and after TTX application. (B) Threshold of Na^+ spikes (as measured at the soma, see Experimental Procedures) evoked in primary (red), intermediate (blue) and terminal (green) branches. (C) dV/dt of Na^+ spikes evoked by clustered (red) or distributed (green; stretch: 50-115 μm) input pattern in the same dendritic segment (p=0.147, Wilcoxon test). (D) Cumulative distribution of Na^+ spike strength in primary parent (black, n=82) and terminal daughter (grey, n=94) basal dendrites of CA3 pyramidal neurons (p<0.001, Kolmogorov-Smirnov test). (E-F) Distance dependent distribution of dendritic Na^+ spike strength in primary parent dendrites (E) and in terminal daughter dendrites (F) in different subregions of CA3. Note that the same dataset was used for general comparison of CA1 and CA3 in Figure 2A-B, except that, because of the uneven number of data points in the three subregions, data from CA3a were reduced by selecting every third datapoint. (G-J) The low ratio of daughter dendrites coupled to strong parent dendrites (Figure 2D) was not a result of the more negative V_{rest} of CA3PCs (CA3: -68.0 ± 0.2 mV, n=381; CA1: -60.0 ± 0.3 mV, n=79, p<0.001, Mann-Whitney test). (G) Stack image of a basal dendritic family with strong parent (P) and connected weak daughter (D) branches. (H) Voltage response (left) and its first temporal derivative (right) evoked by synchronous synaptic stimulation in the strong parent dendrite (dV/dt > 2V/s). (I) Left, representative voltage responses evoked by synchronous synaptic stimulation in the weak daughter dendrite 29 μm distal from the branch point, with (red) and without (black) current injection. Right, rising phase of the traces expanded, with corresponding dV/dt traces on the right. (J) Summary of the effect of depolarization to ~ -60 mV on dV/dt in weak daughter branches. While depolarization increased dV/dt (p<0.05, Wilcoxon test), none of the daughter branches became functionally coupled to the strong parent dendrite even at -60 mV. (K) APs directly triggered by dendritic Na^+ spikes in CA3PCs (n=10) had shorter latency (black) and smaller jitter (gray) than APs generated by the slow component (n=9, p<0.01 for both, Mann-Whitney test), although the latency (but not the jitter) of dendritic spike-evoked APs was even shorter in CA1PCs (latency: p<0.01; jitter: p=0.305, n=7, Mann-Whitney test). Data are represented as mean ± SEM.

You created this PDF from an application that is not licensed to print to novaPDF printer (http://www.novapdf.com)

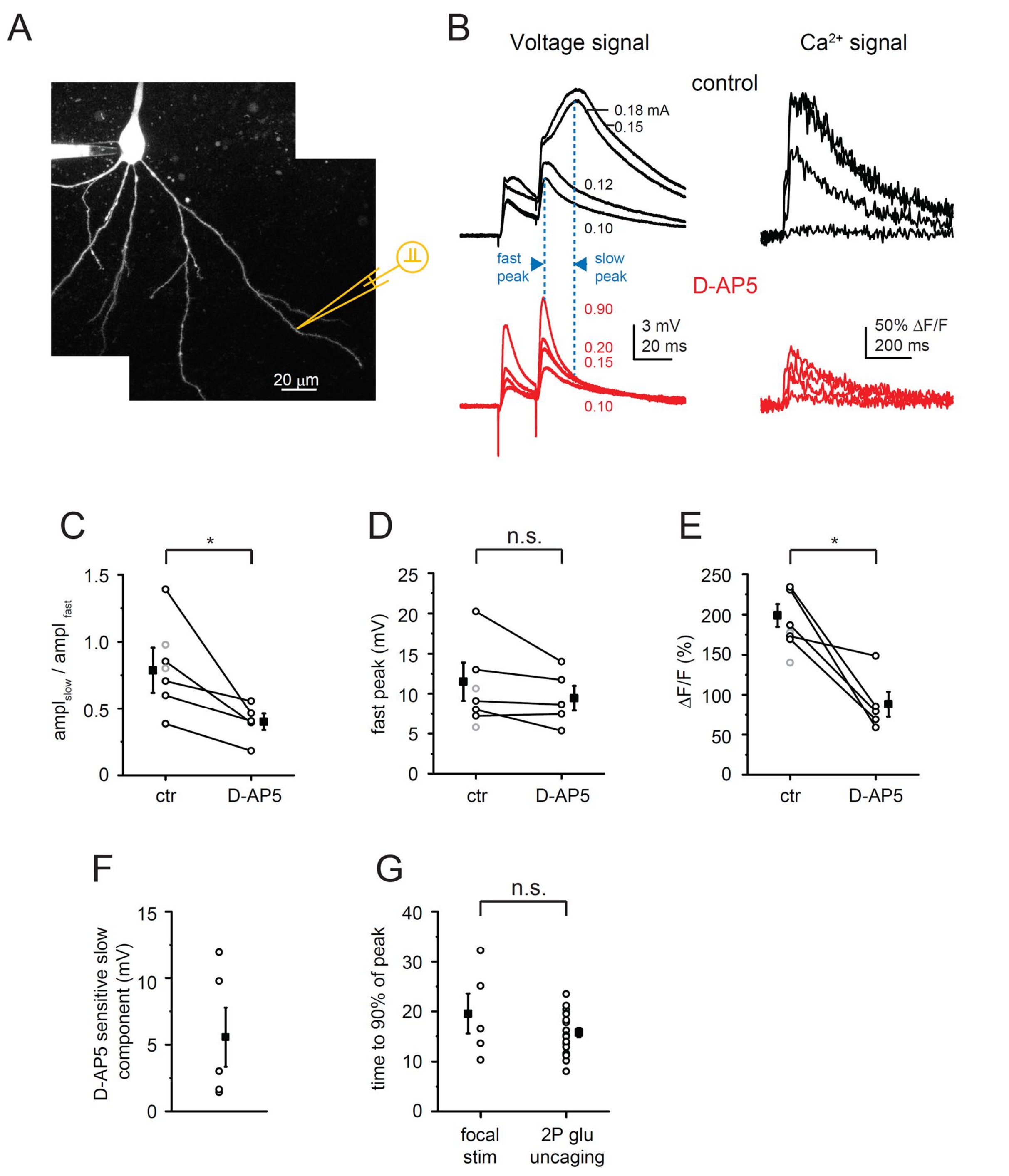

Figure S2. Focal synaptic stimulation of CA3PC basal dendrites, related to Figure 3.

(A) Collapsed z-stack of a CA3PC filled with 100 µM OGB-1 and 50 µM Alexa Fluor 594. Position of the theta pipette used for focal stimulation is indicated in orange. (B) Representative

You created this PDF from an application that is not licensed to print to novaPDF printer (http://www.novapdf.com)

voltage (left) and dendritic Ca^{2+} responses to electrical stimulation under control conditions (black, upper traces) and after 10-minute-long application of 100 µM D-AP5 (red, lower traces). Traces are averages of 3-5 recordings at each stimulus intensity. Stimulus intensity values (in mA) are indicated. Blue dashed lines represent the time points for measurement of the fast peak and slow peak. (C) Effect of D-AP5 on the relative magnitude of the slow component, quantified as the ratio of the slow component amplitude (measured at 20 ms after 2nd stimulus start) and the fast peak amplitude (measured at ~5 ms after 2nd stimulus start). Data were taken at stimulus intensities that generated a slow component under control conditions. Gray open circles represent data from two additional dendrites that were not tested for D-AP5 and were not used for the statistical analysis ($p<0.05$, Wilcoxon test). (D) Effect of D-AP5 on the fast peak ($p=0.079$, Wilcoxon test). (E) Effect of D-AP5 on local dendritic Ca^{2+} signals ($p<0.05$, Wilcoxon test). (F) Magnitude of the D-AP5 sensitive slow component, measured as the average voltage difference of control and D-AP5-treated traces at 20 ms after 2nd stimulus start using suprathreshold stimulation intensities. (G) Time from stimulus onset (focal stim.: 2nd stimulation; 2P uncaging: start of uncaging) to 90% of the peak with the two protocols ($p=0.518$, Mann-Whitney test). Data are represented as mean ± SEM.

We would like to draw attention to differences between focal stimulation (FS) and two-photon glutamate uncaging (2PU): 1) stimulation of local axons is virtually synchronous with FS, in contrast to the input pattern being spread out over several ms with 2PU; 2) therefore fast (presumably AMPA receptor mediated) and slow (NMDAR mediated) responses are temporally more separated with FS than with 2PU, where they overlap more in time; 3) as a consequence, peak responses measured using 2PU may increase more gradually with stronger stimulation (see Fig. 1), rather than strongly saturating; 4) the size of the fast component using FS may be rather independent from that of the slow NMDAR mediated component because it also includes activation of other axons that target the dendritic arbor in a distributed fashion and that do not contribute to generation of the local NMDA spike.

Despite these differences, the time to 90% peak of the D-AP5 sensitive component using FS was remarkably similar to that measured using 2PU (n=20 randomly selected dendrites with 20-30 inputs (Figure S2G), strongly indicating that the activation and time course of the NMDAR mediated component is mechanistically similar with the two stimulation techniques. These results confirm that – although physiological synaptic activity may produce less NMDAR activation - the NMDAR mediated amplification was not solely a product of glutamate uncaging.

You created this PDF from an application that is not licensed to print to novaPDF printer (http://www.novapdf.com)

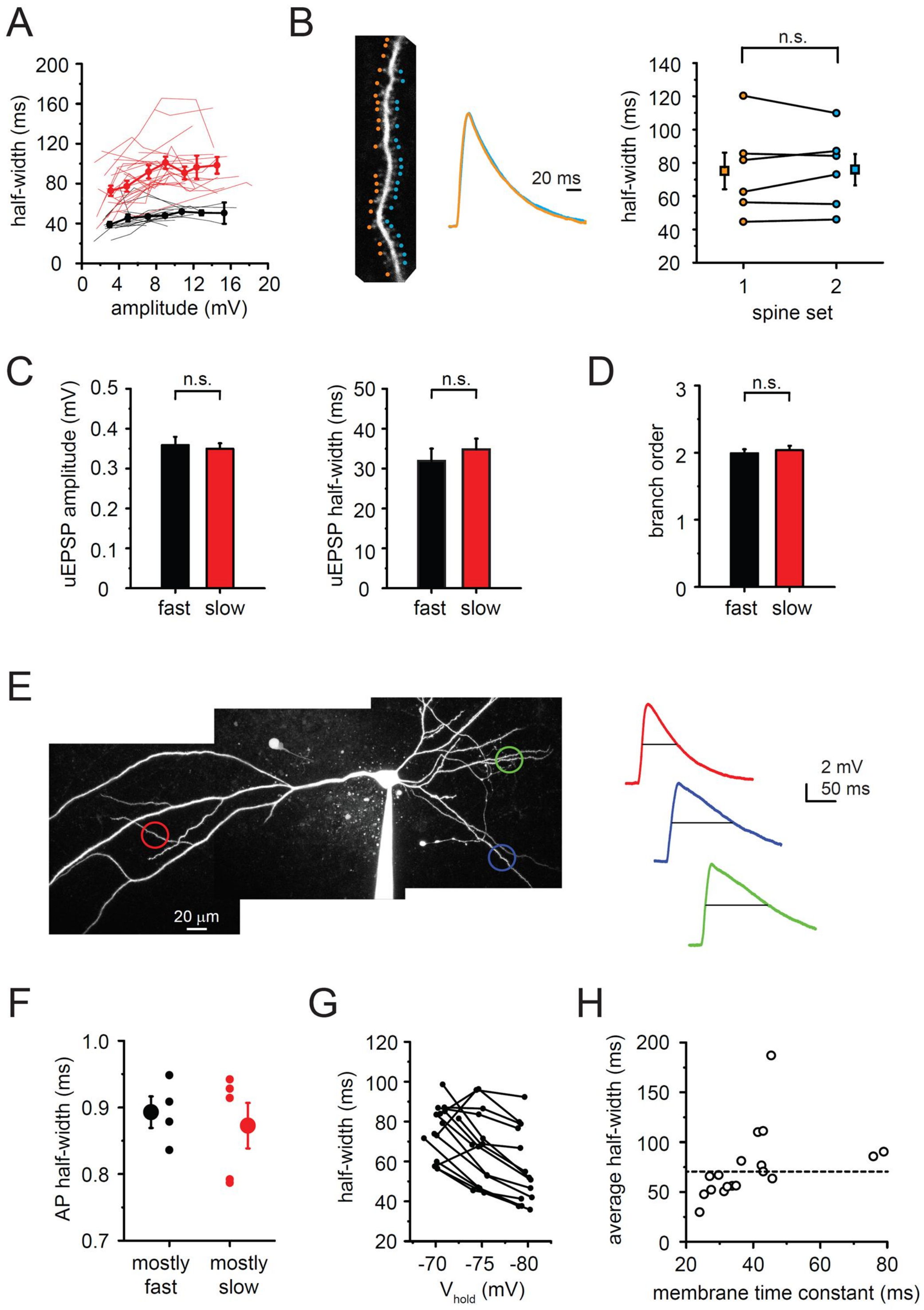

Figure S3. Characteristics of NMDA spike kinetics, related to Figure 5.
(A) The half-width of fast (black) and slow (red) NMDA spikes is constant and can be reliably separated when response peak amplitude exceeds 6 mV. (B) NMDA spike kinetics is

You created this PDF from an application that is not licensed to print to novaPDF printer (http://www.novapdf.com)

characteristic to the dendrite but not to the particular synapses that evoked the spike. Left, dendritic segment with 20 synapses stimulated on either the left or the right side. Middle, normalized voltage responses evoked by left side (orange) and right-side (blue) synapses. Right, summary of results using two non-overlapping sets of stimulated synapses (p=0.753, Wilcoxon test). (C) Amplitude (left, n=19/27 fast/slow, p=0.955, Mann-Whitney test) and half-width (right, n=13/13 fast/slow, p=0.383, Mann-Whitney test) of the unitary EPSP evoked by glutamate uncaging did not differ between fast and slow NMDA spikes. uEPSP kinetics was analyzed in cells with low spontaneous activity. (D) Morphological order of the basal dendritic segments tested was not different between fast (n=146) and slow (n=112) NMDA spikes (p=0.759, Mann-Whitney test). (E) Left, z-stack image of a CA3PC with three stimulated dendrites highlighted in different colors. Right, voltage responses evoked by uncaging in the indicated dendrites. Note the different response half-width in apical and basal dendrites. (F) Action potential half-width was not increased in cells having dendrites with mostly slow NMDA spikes compared to cells having dendrites with mostly fast NMDA spikes (n= 4/5 fast/slow, p=0.903, Mann-Whitney test), suggesting that the NMDA spike kinetics was not determined by global changes in K^+ channel function. (G) Effect of membrane potential on NMDA spike half-width (repeated measures ANOVA, p<0.001). (H) Relationship between the average NMDA spike half-width (averaged from 1-3 dendrites of a given cell) and the membrane time constant. Dashed line represents the cutoff value between fast and slow spikes. Data are represented as mean ± SEM.

You created this PDF from an application that is not licensed to print to novaPDF printer (http://www.novapdf.com)

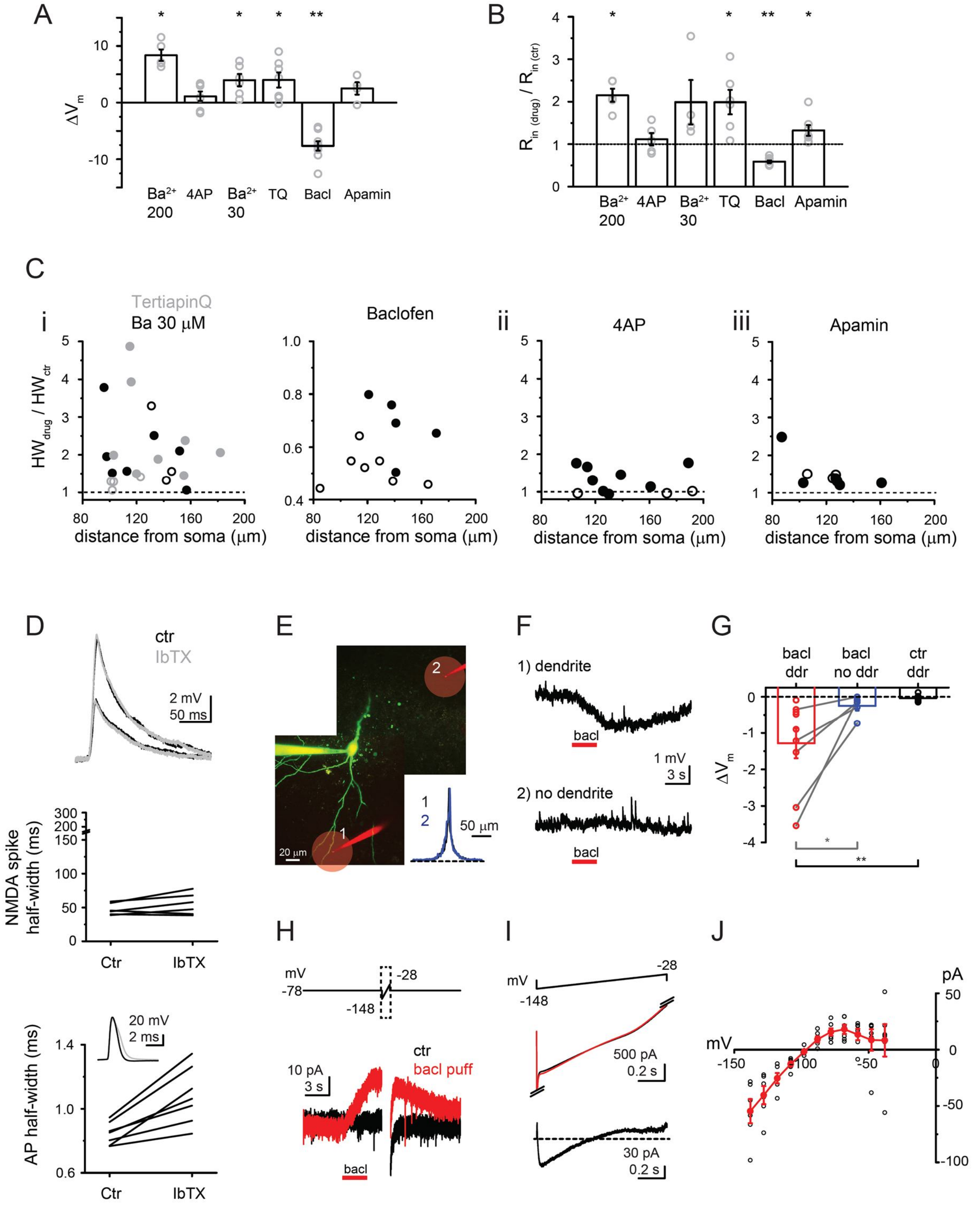

Figure S4. Additional effects of K^+ channel modulators, related to Figure 6.

(A-B) Somatic membrane potential (A, V_m) and input resistance (B, R_{in}) changes in response to bath application of 200 μM Ba^{2+}, 2 mM 4-AP (in the presence of 1 μM TTX), 30 μM Ba^{2+}, 0.5 μM tertiapin-Q (TQ), 20 μM baclofen (Bacl) and 0.1 μM apamin. Changes in V_m were

You created this PDF from an application that is not licensed to print to novaPDF printer (http://www.novapdf.com)

compensated by appropriate adjustment of the DC holding current to keep V_m at ~ -70 mV in the experiments shown in Figure 6 and 7F-G. *, p<0.05; **, p<0.01, Wilcoxon tests).

(C) Distance dependence of the effect of specific K^+ channel modulators on half-width (HW). No significant correlation was found for the effect of (i) GIRK inhibitors 0.5 μM tertiapinQ (Spearman R=-0.238, p=0.582) and 30 μM Ba^{2+} (Spearman R=-0.428, p=0.353) on fast NMDA spike half-width (left) and the $GABA_B$ agonist GIRK activator 20 μM baclofen (right; Spearman R=0.112, p=0.906; both fast and slow spikes included) with distance from soma. No correlation with distance was found for the effect of (ii) 2 mM 4-AP (in the presence of 1 μM TTX; Spearman R=-0.071, p=0.881) and (iii) 0.1 μM apamin (Spearman R=-0.738, p=0.057) on fast NMDA spike half-width. Filled circles represent fast spikes under control conditions; open circles represent slow spikes under control conditions. (D) The BK channel inhibitor iberiotoxin (IbTX, 0.1 μM) had no effect on half width of fast NMDA spikes. Top, representative individual traces at two stimulation strengths under control conditions (black) and after application of IbTX (gray). Middle, summary data (p=0.115, Wilcoxon test). Lower, IbTX increased the halfwidth of somatic APs (p<0.05, Wilcoxon test; inset: representative traces in control (black) and IbTX (gray)).

(E-J) GIRK current in distal dendrites of CA3PCs.
E-G) Current clamp experiments. E) Stack image of a CA3PC loaded with Alexa Fluor 488 and the focal puffer pipette loaded with 100 μM baclofen and 50 μM Alexa Fluor 594, positioned at two different locations (1, puffing at a dendrite 148 μm from the soma; 2, puffing at a dendrite-free region at a similar distance from soma and dendrites). Red circles illustrate spatial dimension of the Alexa Fluor 594 fluorescence at the end of the 3-sec-long puffing. Apical dendrite of the cell heads deep down into the slice. Inset: spatial profile of puffing, indicated by the normalized fluorescence profile of Alexa Fluor 594 at the end of puffing in location 1 (black) and 2 (blue). F) Representative somatic voltage response to 3-sec-long baclofen puff at location 1 (upper panel) and location 2 (lower panel) shown in E. V_m at rest was held at -70 mV. G) Summary of the effect of baclofen (measured at 5-6 sec after onset of application) puffed onto dendrites (red, n=9, distance from soma: 149 ± 8 μm) and at dendrite-free locations (blue, n=5, p<0.05 compared to dendritic puff in the same cells (gray lines), Wilcoxon test; distance from closest dendrite/soma: 139 ± 10 μm). No V_m change was observed when puffing baclofen-free puffer solution at dendrites (ctr, black, n=6, p<0.01 compared to dendritic baclofen puff, Mann-Whitney test; distance from soma: 150 ± 7 μm). H-J) Voltage clamp experiments. H) Upper panel, voltage command protocol; lower panel, representative recording puffing baclofen (100 μM for 3 sec) at a dendrite 137 μm from the soma (red, average of 10 traces). Intermittent with recordings of baclofen puffing, control traces (same protocol, no pressure applied to puffer) were recorded (black, average of 10 traces). Voltage dependence of the baclofen activated current was tested using a slow, 1-sec-long ramp from -148 to -28 mV, beginning at 5 sec after the onset of puffing. Voltage commands were corrected for the measured liquid junction potential (8 mV). The steady-state nature of the baclofen-activated current and the slow ramp used make somatic recordings interpretable in these experiments despite the distance of the activated region from the soma. Currents during the ramp are blanked for clarity and are shown enlarged in I. I) Ramp section of the representative recording shown in H. Upper panel, voltage command protocol. Middle panel, current traces during the ramp. Black, control (no pressure applied); red, during baclofen puffing. Lower panel, baclofen induced current, obtained by subtracting the traces in middle panel. Dashed line represents zero current. J) Summarized I-V plot using data from 6 dendrites in 6 cells

You created this PDF from an application that is not licensed to print to novaPDF printer (http://www.novapdf.com)

(distance from soma: 145 ± 8 μm). Currents were averaged in ±1 mV bins around the mean voltage. The baclofen-activated current reversed at -96.1 ± 1.3 mV (n=6), close to the equilibrium potential of K^+ (-101.4 mV). The baclofen-activated current at -78 mV was similar in steady-state and ramp conditions (16.7 ± 4.1 pA and 15.7 ± 3.4 pA, respectively; n=6, p=0.248, Wilcoxon test). Data are represented as mean ± SEM.

You created this PDF from an application that is not licensed to print to novaPDF printer (http://www.novapdf.com)

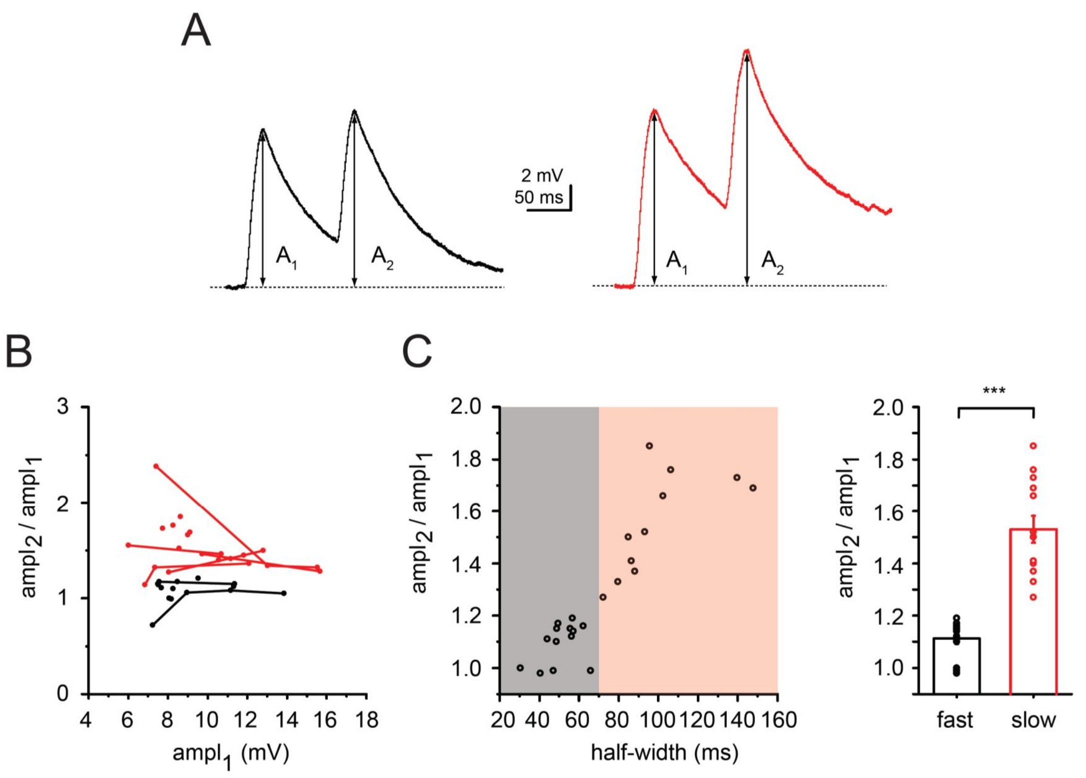

Figure S5. Paired pulse ratio of NMDA spikes, related to Figure 7.
(A) Representative voltage responses to paired stimulation with 100 ms interval by fast (left, black) and slow (right, red) NMDA spikes. (B) The paired pulse amplitude ratio was characteristically stable in a wide amplitude range. (C) Left, strong correlation between paired pulse amplitude ratio and half-width (Spearman R=0.899, $p<0.001$). Gray and red shades indicate fast and slow NMDA spike ranges, respectively. Right, paired pulse amplitude ratio of fast vs. slow NMDA spikes ($p<0.001$, Mann-Whitney test). Data are represented as mean ± SEM.

You created this PDF from an application that is not licensed to print to novaPDF printer (http://www.novapdf.com)

Supplemental Experimental Procedures

Slice preparation

Adult male Sprague-Dawley rats (8-12-week-old) were used to prepare transverse slices (400 μm) from the hippocampus similarly to that described previously (Losonczy and Magee, 2006), according to methods approved by the Janelia Farm Institutional Animal Care and Use Committee and by the Animal Care and Use Committe (ACUC) of the Institute of Experimental Medicine, Hungarian Academy of Sciences, and was in accordance with 86/609/EEC/2 and DIRECTIVE 2010/63/EU Directives of the European Community. Animals were deeply anaesthetized with 5% isoflurane and quickly perfused through the heart with ice-cold cutting solution containing (in mM): sucrose 220, $NaHCO_3$ 28, KCl 2.5, NaH_2PO_4 1.25, $CaCl_2$ 0.5, $MgCl_2$ 7, glucose 7, Na-pyruvate 3, and ascorbic acid 1, saturated with 95 % O_2 and 5 % CO_2. The brain was quickly removed and slices were prepared in cutting solution using a vibratome (Vibratome, St. Louis, MO, or Leica VT1000A, Leica Biosystems GmbH, Nussloch, Germany). Slices were incubated in a submerged holding chamber in ACSF at 35 °C for 30 min and then stored in the same chamber at room temperature.

Characterization of dendritic Na^+ spikes

The strength of Na^+ spikes was determined by the peak of the first temporal derivative (dV/dt) of voltage traces. Strong Na^+ spikes were defined as dV/dt > 2V/s based on CA1 data (Losonczy et al., 2008; Makara et al., 2009). dV/dt values that could not be measured reliably from noise (usually data smaller than 0.2 V/s) were considered as 0. To determine Na^+ spike threshold (as measured at the soma) at a given dendritic location, the uncaging laser power was adjusted to the lowest level that was sufficient to evoke the spike with synchronous uncaging, and the same power was used to record the individual spine responses (with 200-210 ms interval) in the same experimental session. Spike threshold was then calculated as the arithmetic sum of the unitary gluEPSPs. Data for dV/dt distribution of Na^+ spikes in CA1 pyramidal neurons partly included results used in the control population of our temporally overlapping previous study (Makara et al., 2009), as well as new experiments.

Characterization of NMDA spikes

In experiments where NMDAR-mediated nonlinearity was quantified by determining the expected vs. measured amplitude, we used a large number (20-40) of closely clustered, distal-to-proximal arranged inputs, with unitary gluEPSP amplitude and rise time close to that of mEPSPs as measured by sucrose application at dendritic segments 70-168 μm from the soma as described before (Magee and Cook, 2000; mEPSP amplitude: 0.36 ± 0.06 mV, RT20-80: 1.93 ± 0.14 ms, n=5 puffing points in 3 cells; uEPSPs, amplitude: 0.36 ± 0.01 mV, n=98, p=0.228; RT20-80: 2.08 ± 0.08 ms, measured in n=26 cells with low spontaneous activity, p=0.376). mEPSPs were analyzed using EVAN (Nusser et al., 2001).

Unitary gluEPSPs were measured repeatedly (usually 2-5 times) interleaved with synchronous stimulations, using 205-420 ms intervals between the individual synapses. To standardize these experiments, results were included in the analysis only if 1) at least two-third of the selected spines demonstrated measurable gluEPSP, 2) the average amplitude of the successful unitary gluEPSPs was 0.2-0.6 mV and maximum unitary gluEPSP amplitude was ≤ 1.2 mV, 3) at

You created this PDF from an application that is not licensed to print to novaPDF printer (http://www.novapdf.com)

least 5 mV expected amplitude was achieved, and 4) unitary responses were stable with repeated stimulation. When constructing expected vs measured amplitude graphs, data from all dendrites were usually sorted into 1 mV bins.

To determine the half-width value of NMDA spike of a given dendrite, values from responses with peak amplitude over 6 mV were averaged (see Figure S3A). For paired pulse (Figure 7, Figure S5) and pharmacological experiments on half-width (Figure 6), we used ≥ 20 inputs and input-output relationship was usually not determined. In experiments where the effect of K^+ channel inhibitors on paired-pulse ratio was tested, all traces where the amplitude of the first pulse response exceeded 6 mV were analyzed (see Figure S5B).

Focal electrical stimulation (Figure S2)

Focal electrical synaptic stimulation (two 0.1 ms pulses at 50 Hz, BioStim stimulator system, Supertech Ltd, Pecs, Hungary) was performed using silver wires inserted into a theta pipette filled with ACSF containing 2 mM $CaCl_2$ (see solutions above). The tip of the theta pipette was placed in close proximity (~2-3 µm) to a basal dendritic segment, guided by the fluorescent image of the dendrite overlaid with two-photon Dodt image visualizing the theta pipette. For these experiments the cells were loaded with the Ca^{2+} sensitive dye Oregon Green BAPTA-1 (OGB-1, 100 µM) and Alexa Fluor 594 (50 µM). Ca^{2+} signals were measured in linescan mode at 233 Hz. Only those dendrites where peak voltage responses at the soma exceeded 6 mV and local dendritic Ca^{2+} signals exceeded 130% $\Delta F/F$ were included in the analysis.

Focal baclofen application (Figure S4E-J)

For focal baclofen application (puffing) experiments the bath ACSF was complemented with 1 µM tetrodotoxin to reduce spontaneous network activity. A regular patch pipette containing 100 µM baclofen and 50 µM Alexa Fluor 594 (dissolved in the above bath solution) served as a puffer pipette. Higher concentration of baclofen was used due to the expected lower effective concentration achieved by this application method. The puffer pipette was guided to close proximity (~5 µm) of a dendritic segment (identified by intracellular Alexa Fluor 488), using overlaid Dodt contrast imaging. Pressure was applied for 3 seconds via a pneumatic ejection system (PDES-02TE, NPI). The size of the puffed area was monitored by measuring fluorescence of Alexa Fluor 594 in linescan mode around the tip of the puffer pipette. Pressure was adjusted to produce an area with an estimated 30-65 µm diameter (10 % of peak Alexa Fluor 594 fluorescence) at the end of puffing.

Morphological analysis

The proximodistal position of CA3 neurons was determined by their localization as revealed by the Alexa Fluor 488 or 594 fluorescence or biocytin staining. The three sub-fields of CA3 were categorized similar to previous studies (Lorente de No, 1934; Li et al., 1994). The midline of the fimbria was considered as the border between CA3a and CA3b, and the section between and ~200 µm distal to the blades of the dentate gyrus was considered as CA3c. All neurons had their somata in CA3 either in stratum pyramidale or in the adjacent border of stratum oriens, had pyramidal morphology with dendrites extending only within CA3 (not to the hilus in

You created this PDF from an application that is not licensed to print to novaPDF printer (http://www.novapdf.com)

CA3c) and often intact local axon collaterals innervating CA3, and had spines and perisomatic thorny excrescences, confirming that they were CA3 pyramidal neurons.

Supplemental References

Li, X.G., Somogyi, P., Ylinen, A., and Buzsáki, G. (1994). The hippocampal CA3 network: an in vivo intracellular labeling study. J. Comp. Neurol. *339*, 181-208.

Lorente De No, R. (1934). Studies on the structure of the cerebral cortex. Continuation of the study of the ammonic system. J. Psychol. Neurol. *46*, 113–177.

Losonczy, A., and Magee, J.C. (2006). Integrative properties of radial oblique dendrites in hippocampal CA1 pyramidal neurons. Neuron *50*, 291-307.

Losonczy, A., Makara, J.K., and Magee, J.C. (2008). Compartmentalized dendritic plasticity and input feature storage in neurons. Nature *452*, 436-441.

Magee, J.C., and Cook, E.P. (2000). Somatic EPSP amplitude is independent of synapse location in hippocampal pyramidal neurons. Nat. Neurosci. *3*, 895-903.

Makara, J.K., Losonczy, A., Wen, Q., and Magee, J.C. (2009). Experience-dependent compartmentalized dendritic plasticity in rat hippocampal CA1 pyramidal neurons. Nat. Neurosci. *12*, 1485-1487.

Nusser, Z., Naylor, D., and Mody, I. (2001). Synapse-specific contribution of the variation of transmitter concentration to the decay of inhibitory postsynaptic currents. Biophys. J. *80*, 1251-1261.

You created this PDF from an application that is not licensed to print to novaPDF printer (http://www.novapdf.com)
